# Sleep Duration and the Risk of Metabolic Syndrome in Adults: A Systematic Review and Meta-Analysis

**DOI:** 10.3389/fneur.2021.635564

**Published:** 2021-02-18

**Authors:** Jianian Hua, Hezi Jiang, Hui Wang, Qi Fang

**Affiliations:** ^1^Department of Neurology, The First Affiliated Hospital of Soochow University, Suzhou, China; ^2^Medical College of Soochow University, Suzhou, China; ^3^Department of Cardiology, The First Affiliated Hospital of Soochow University, Suzhou, China

**Keywords:** sleep duration, metabolic syndrome, cohort study, meta-regression, meta-analysis

## Abstract

**Objective:** Epidemiological studies have reported inconsistent findings for the association between sleep duration and metabolic syndrome. We aimed to clarify the effects of short and long sleep durations on metabolic syndrome in adults by performing a meta-analysis.

**Methods:** Adopting random-effects models, this study analyzed the effects of short and long sleep durations based on data from prospective cohort studies and cross-sectional studies retrieved from four electronic databases from inception to May 2020.

**Results:** We collected data from 235,895 participants included in nine prospective cohort studies and 340,492 participants included in 27 cross-sectional studies. In cohort studies, short sleep duration was associated with an increased risk of metabolic syndrome (RR, 1.15; 95% CI, 1.05–1.25, *I*^2^ = 63.1%, *P* < 0.001) compared with normal sleep duration. While long sleep duration was not associated with new-onset metabolic syndrome (RR, 1.02, 0.85–1.18, *I*^2^ = 38.0%, *P* = 0.491). In cross-sectional studies, both short (OR, 1.06, 95% CI, 1.01–1.11, *I*^2^ = 66.5%, *P* < 0.001) and long (OR, 1.11, 95% CI, 1.04–1.17, *I*^2^ = 73.8%, *P* < 0.001) sleep durations were associated with a high prevalence of metabolic syndrome.

**Conclusions:** Only a short sleep duration was associated with an increased risk of metabolic syndrome. Future studies should address whether the association is casual and modifiable.

## Introduction

Metabolic syndrome (MetS) is a cluster of disorders that occur together, including central obesity, hypertension, increased fasting glucose levels, higher triglyceride (TG) levels, or low high-density cholesterol (HDL) levels. The National Cholesterol Education Program's Adult Treatment Panel III (NECP ATP-III), the American Heart Association/National Heart Lung and Blood Institute (AHA-NHLBI), and other organizations have issued their own definitions for this syndrome. The prevalence of metabolic syndrome ranges from 20 to 45% in the population (1) and from 20 to 30% among different ethnicities in the United States (2) and is ~24% in Asia (3, 4). Metabolic syndrome is associated with adverse cardiovascular events, even after adjusting for diabetes and obesity (5, 6). It not only imposes a strain on global health but also imposes a financial burden on patients and the health system due to the need for multiple medications (7). Therefore, the modifiable risk factors for metabolic syndrome must be identified (8).

Short and long sleep durations are known to increase the risk of serious health outcomes, including diabetes, cardiovascular disease, and mortality (9, 10), which have strong associations with metabolic syndrome (5, 6). Several meta-analyses have examined the association between sleep duration and metabolic syndrome (11–13) and reported mixed results. Nevertheless, in the primary results of the previous studies, ORs and HRs were pooled together, whereas they were not statistically interchangeable in our study. First, the OR provides a snapshot of the association at a certain time point, while HR takes into account both the number and timing of event occurrence (14). Studies assessing the association using a prospective cohort design have less substantial bias and might provide stronger support for causality (10, 15, 16). Second, the prevalence of metabolic syndrome is >20%. The RR is difficult to estimate from the OR (17).

By the time *Ju* conducted a meta-analysis in 2013, two cohort studies assessing the effects of short sleep duration, and only one cohort study examining the effects of a long sleep duration had been published (11). Moreover, many articles were published after the completion of the previous meta-analysis, necessitating an update of the overall association. Therefore, we conducted a systemic review and meta-analysis to (1) examine the association between short/long sleep duration and metabolic syndrome in adults compared with moderate sleep duration and (2) assess prospective cohort studies and cross-sectional studies separately.

## Materials and Methods

We performed this study according to Preferred Reporting Items for Systematic Reviews and Meta-Analysis (PRISMA) guidelines (Table 1).

**Table 1:** PRISMA 2009 checklist.

**Section/topic**	**#**	**Checklist item**	**Reported on page #**
**Title**			
Title	1	Identify the report as a systematic review, meta-analysis, or both.	1
**Abstract**			
Structured summary	2	Provide a structured summary including the following information as applicable: background; objectives; data sources; study eligibility criteria, participants, and interventions; study appraisal and synthesis methods; results; limitations; conclusions and implications of key findings; systematic review registration number.	2
**Introduction**			
Rationale	3	Describe the rationale for the review in the context of what is already known.	3
Objectives	4	Provide an explicit statement of questions being addressed with reference to participants, interventions, comparisons, outcomes, and study design (PICOS).	3-4
**Methods**			
Protocol and registration	5	Indicate if a review protocol exists, if and where it can be accessed (e.g., Web address), and, if available, provide registration information including registration number.	NA
Eligibility criteria	6	Specify study characteristics (e.g., PICOS and length of follow-up) and report characteristics (e.g., years considered, language, and publication status) used as criteria for eligibility, giving the rationale.	4
Information sources	7	Describe all information sources (e.g., databases with dates of coverage, contact study authors to identify additional studies) in the search and date last searched.	4
Search	8	Present full electronic search strategy for at least one database, including any limits used, such that it could be repeated.	4, Supplementary Appendix 1
Study selection	9	State the process for selecting studies (i.e., screening, eligibility, included in systematic review, and, if applicable, included in the meta-analysis).	3-4
Data collection process	10	Describe the method of data extraction from reports (e.g., piloted forms, independently, or in duplicate) and any processes for obtaining and confirming data from investigators.	4-5
Data items	11	List and define all variables for which data were sought (e.g., PICOS and funding sources) and any assumptions and simplifications made.	4-5
Risk of bias in individual studies	12	Describe methods used for assessing risk of bias of individual studies (including specification of whether this procedure was performed at the study or outcome level), and how this information is to be used in any data synthesis.	5
Summary measures	13	State the principal summary measures (e.g., risk ratio and difference in means).	5
Synthesis of results	14	Describe the methods of handling data and combining results of studies, if conducted, including measures of consistency (e.g., *I*^2^) for each meta-analysis.	5
Risk of bias across studies	15	Specify any assessment of risk of bias that may affect the cumulative evidence (e.g., publication bias and selective reporting within studies).	5
Additional analyses	16	Describe methods of additional analyses (e.g., sensitivity or subgroup analyses, meta-regression analysis), if conducted, indicating which were pre-specified.	5-6
**Results**			
Study selection	17	Provide the numbers of studies screened, assessed for eligibility, and included in the review, with reasons for exclusion at each stage, ideally with a flow diagram.	6, Figure 1
Study characteristics	18	For each study, present characteristics for which data were extracted (e.g., study size, PICOS, and follow-up period) and provide the citations.	6, Tables 2, 3
Risk of bias within studies	19	Present data on risk of bias of each study and, if available, any outcome level assessment (see item 12).	6, Tables 2, 3
Results of individual studies	20	For all outcomes considered (benefits or harms), present for each study: (a) simple summary data for each intervention group (b) effect estimates and confidence intervals, ideally in a forest plot.	Tables 2, 3, Figures 2–5
Synthesis of results	21	Present the results of each meta-analysis conducted, including confidence intervals and measures of consistency.	6-7
Risk of bias across studies	22	Present the results of any assessment of risk of bias across studies (see Item 15).	7
Additional analysis	23	Present the results of additional analyses, if performed (e.g., sensitivity or subgroup analyses, meta-regression analysis [see Item 16]).	7-8, Tables 4, 5
**Discussion**			
Summary of evidence	24	Summarize the main findings including the strength of evidence for each main outcome; consider their relevance to key groups (e.g., healthcare providers, users, and policy makers).	8
Limitations	25	Discuss limitations at study and outcome levels (e.g., risk of bias), and at the review level (e.g., incomplete retrieval of identified research and reporting bias).	9-10
Conclusions	26	Provide a general interpretation of the results in the context of other evidence, and implications for future research.	10
**Funding**			
Funding	27	Describe sources of funding for the systematic review and other support (e.g., supply of data); role of funders in the systematic review.	10

*Moher et al. (76)*.

Two independent researchers (JNH and HZJ) separately assessed the eligibility, extracted data, and assessed the quality of the included studies. Any disagreement in screening the articles was resolved through discussion between these two investigators, with adjudication by a third researcher (QF) if disagreements persisted.

### Search Strategy

A systematic search strategy was employed to identify all articles published from database inception to May 2020. Articles were identified through searches of Medline, Embase, CINAHL, and PsycINFO. The search terms for each database are shown in Supplementary Appendix 1. This strategy combined terms characterizing metabolic syndrome as the outcome variable and sleep duration as the exposure variable. The considered articles were not limited to English-language articles. We also screened conference proceedings, journals, and reference lists of included studies and previous systemic reviews.

### Selection Criteria

We used the following PICOS criteria (population, intervention, control, comparison, outcome, study) to define the selection criteria.

- P: For prospective cohort studies, the study population was adults without metabolic syndrome at baseline. For cross-sectional studies, the population was adults.
- I: Individuals with short or long sleep duration.
- C: Individuals with moderate sleep duration.
- O: Metabolic syndrome.
- S: Prospective cohort studies or cross-sectional studies.

**Figure 1:**
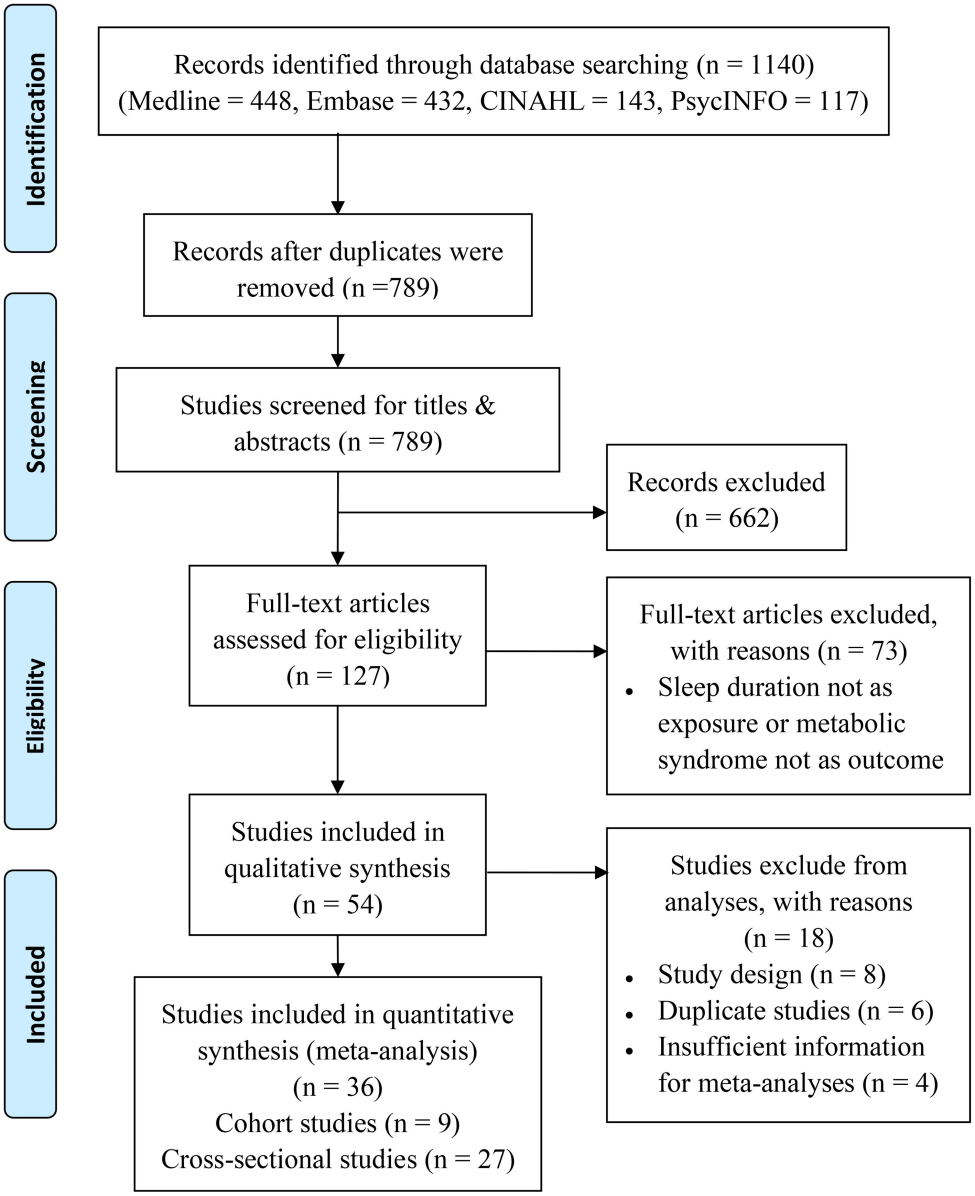
Flowchart for the included studies.

If multiple articles reported associations based on the same cohort, only the article with the largest sample size was included. The inclusion of studies was conducted in two stages: (1) screening of the title and abstract and (2) screening of the full text (Figure 1).

### Data Extraction

The following information was extracted from each eligible study: author name and publication year, study type, study location (country and continent), sample size, participant characteristics (age range, mean age, and sex composition), exposure and outcome measurements (sleep measurement, metabolic syndrome measurement/diagnostic criteria for metabolic syndrome, and definition of long or short sleep duration), and main results.

Since the definition of sleep duration varies among studies (18), the three categories of sleep duration (short, long, and moderate) were extracted in one of two ways. For some papers, the author had already divided the sleep duration into three categories based on cultures and ethnicities. For others in which sleep duration was divided into more than three groups, short or long sleep duration was defined as the shortest or longest range reported in the article (10). The midpoint of the categories was defined as the moderate sleep duration range. Regarding the main results, the adjusted estimates that reflected the most comprehensive control were extracted. Sleep measurement is the method used to assess sleep duration, such as questionnaires, interviews (self-reported), and polysomnography (objective).

Data were extracted by two investigators (JNH and HZJ) independently. Any disagreement in screening the articles was resolved by discussion between the two investigators. Consultation with a third investigator (QF) was performed if necessary.

### Exposure and Outcome Measures

Regarding the measurement of sleep duration, two studies used objective measurements, while others used interviews or questionnaires.

The diagnostic criteria of metabolic syndrome varied between studies. Ten studies used the Third Report of the National Cholesterol Education Program's Adult Treatment Panel III (NECP ATP-III), four studies used the modified NECP ATP-III, 14 studies used the American Heart Association/National Heart Lung and Blood Institute (AHA-NHLBI), three studies used the International Diabetes Federation (IDF) (12), and five studies used other criteria.

### Quality Appraisal

The quality of all studies was evaluated using the Newcastle-Ottawa Quality Assessment Scale (NOS) (19). The total score ranged from 0 to 9 points. For the outcome category and comparability category, all the studies had a similar quality. The difference between studies lies in the study design category (Supplementary Table 1).

### Data Analysis

We conducted all the analyses described below separately for cohort studies and cross-sectional studies and for study-specific short and long sleep durations.

In the analysis of cohort studies, hazard ratios (HRs) were regarded as risk ratios (RRs). For studies that provided only odds ratios (ORs), we calculated RRs using the ORs and control event rates (CERs) in individuals with moderate sleep durations. Using random-effect models, we estimated the pooled RR and 95% CI. For cross-sectional studies, we calculated the pooled OR and 95% CI using random-effect models.

Heterogeneity between the studies was assessed using Cochran Q statistics (*P* < 0.1 indicates statistically significant heterogeneity) and *I*^2^ statistics (*I*^2^ > 50% indicates statistically significant heterogeneity) (20). We used a funnel plot, Egger's regression test, and the Begg and Mazumdar test to examine publication bias (21, 22). The “trim and fill” method was used to adjust the funnel plot and recalculate the results (23, 24). The sensitivity analysis was performed by sequentially excluding each study to test the robustness of the pooled estimates.

A subgroup analysis was conducted to explore the potential heterogeneity among cross-sectional studies after stratification according to sex, geographic region, the methods used to measure sleep duration, the definitions of short or long sleep duration and metabolic syndrome, study population, sample size, and study quality. We used the z test to compare the pooled estimates of each subgroup (25). Univariate and multivariate meta-regression analyses were conducted to study the effect of possible influential confounders, including the mean age, proportion of males, definition of sleep duration, sample size, and study quality. For cohort studies, subgroup and meta-regression analyses were not performed due to the small number of datasets included in the meta-analysis.

All statistical analyses were performed using Stata 15.1 (Stata Corp, College Station, TX) and the “metafor” package in R-3.4.3 (24). R was used to perform the subgroup analysis and “trim and fill” analysis.

## Results

### Search Results

The initial electronic search yielded 1,140 articles, among which 789 were reviewed based on the title and abstract. A total of 127 full-text articles were retrieved, and 36 studies were included in the final analysis (Figure 1).

### Characteristics of Study Samples

We identified nine prospective cohort studies that examined the association between sleep duration and the incident risk of metabolic syndrome in 235,895 participants. The sample size ranged from 293 to 162,121. The mean follow-up duration ranged from 2 to 8 years (Table 2).

**Table 2:** Characteristics of cohort studies.

**References**	**Study type (follow-up year)**	**Country, Continent**	**Sample size**	**Mean age ± SD, range**	**% Male**	**Study population**	**Sleep measurement**	**Metabolic syndrome measurement**	**Sleep (h)**	**Main findings reported in original articles: Adjusted HR/RR/OR (95% CI)**
Choi et al. (41) (male)	Cohort (2-4)	Korea, Asia	2,093	40–55	0	Community	Interview	NECP ATP-III	<6 6-8 ≥10	aHR 1.80 (1.06–3.05) Ref. aHR 1.57 (0.61–4.01)
Choi et al. (41) (female)	Cohort (2-4)	Korea, Asia	2,133	40–55	100	Community	Interview	NECP ATP-III	<6 6-8 ≥10	aHR 0.62 (0.24–1.64) Ref. aHR 1.66 (0.71–3.88)
Otsuka et al. (28)	Cohort (3.7)	Japan, Asia	2,090	44.6 35-63	47.2	Community	Questionnaire	Japanese Criteria	≤5 >6	aHR 3.18 (1.52–6.64) Ref.
Chaput et al. (42)	Cohort (6)	Canada, North America	293	39.2 ± 14.3 18–65	NA	Community	Questionnaire	AHA-NHLBI	≤6 7-8 ≥9	aHR 1.82 (1.16–4.79) Ref. aHR 1.13 (0.58–1.98)
Kim et al. (43)	Cohort (2.6)	Korea, Asia	2,579	54.1 ± 8.3 ≥20	34.5	Company or office	Interview	AHA-NHLBI	<6 7-8 ≥10	aOR 1.41 (1.06–1.88) Ref. aOR 0.68 (0.39–1.17)
Li et al. (44) (male)	Cohort (4.4)	China, Asia	4,774 for all	30–65	100	Community	Questionnaire	AHA-NHLBI	<6 7-8 >8	aRR 1.87 (1.51–2.30) Ref. aRR 1.96 (1.35–2.85)
Li et al. (44) (female)	Cohort (4.4)	China, Asia	4,774 for all	30–65	0	Community	Questionnaire	AHA-NHLBI	<6 7-8 >8	aRR 0.93 (0.73–1.19) Ref. aRR 0.93 (0.60–1.53)
Song et al., (45)	Cohort (2)	China, Asia	11,661	47.0 ± 12.0 18–98	82.1	Hospital	Questionnaire	AHA-NHLBI	≤5.5 7 ≥8.5	aHR 1.22 (1.00–1.49) Ref. aHR 1.24 (0.93–1.66)
Deng et al. (46)	Cohort (8)	Taiwan, Asia	162,121	20–80	47.4	Community	Questionnaire	AHA-NHLBI	<6 6-8 >8	aHR 1.09 (1.05–1.13) Ref. aHR 0.93 (0.88–0.99)
Itani et al. (47)	Cohort (7)	Japan, Asia	39,182	42.4 ± 9.8 ≥20	100	Company or office	Questionnaire	Japanese Criteria	<5 ≥5	aHR 1.08 (1.03–1.14) Ref.
Yingnan et al. (48)	Cohort (3)	China, Asia	8,969	56.7 ± 7.7 35–75	35	Community	Questionnaire	Chinese Criteria	<6 7-8 >9	aOR 1.25 (0.75–2.08) Ref. aOR 0.96 (0.69–1.33)

Another 27 studies were cross-sectional studies, including 340,492 individuals. The sample size ranged from 263 to 88,678 (Table 3).

**Table 3:** Characteristics of cross-sectional studies.

**References**	**Study type**	**Country/Area, Continent**	**Sample size**	**Mean age ± SD, range**	**% Male**	**Study population**	**Sleep measurement**	**Metabolic syndrome measurement**	**Sleep (h)**	**Main findings reported in original articles: Adjusted HR/RR/OR (95% CI)**
Santos et al. (49) (male)	Cross-sectional	Portugal, Europe	832	18–92	100	Community	Interview	NECP ATP-III	≤6 7 ≥9	aOR 1.40 (0.76-2.60) Ref. aOR 1.50 (0.50-2.60)
Santos et al. (49) (female)	Cross-sectional	Portugal, Europe	1,332	18–92	0	Community	Interview	NECP ATP-III	≤6 7 ≥9	aOR 0.92 (0.55-1.50) Ref. aOR 2.00 (1.30-3.00)
Choi et al. (50)	Cross-sectional	Korea, Asia	4,222	44.1 ± 0.4 ≥20	43.2	Community	Questionnaire	Modified NECP ATP-III	≤5 7 ≥9	aOR 1.17 (0.87-1.59) Ref. aOR1.69 (1.17,2.45)
Hall et al. (51)	Cross-sectional	USA, North America	1,214	44.4 ± 6.830-54	46.6	Community	Interview	AHA-NHLBI	<6 7 >8	aOR 1.76 (1.13-2.74) Ref. aOR 1.69 (0.91-3.00)
Aroar et al. (52)	Cross-sectional	China, Asia	29,333	61.6 ± 7.1 >50	65.1	Community	Interview	Modified NECP ATP-III	<6 7 ≥9	aOR 0.97 (0.88-1.06) Ref. aOR 1.21 (1.20-1.34)
Kobayashi et al. (53)	Cross-sectional	Japan, Asia	44,452	44.8 ± 12.8	49.4	Hospital	Questionnaire	Japanese criteria 2008	<6 7-8 ≥8	aOR 1.40 (1.21-1.60) Ref. aOR 0.98 (0.82-1.20)
Najafian et al. (54)	Cross-sectional	Iran, Asia	12,322	38.8 ± 14.9 >19	48.2	Hospital	Interview	NECP ATP-III	≤5 6 ≥9	aOR 1.52 (1.33-1.74) Ref. aOR 0.79 (0.68,0.94)
McCanlies et al. (55)	Cross-sectional	USA, North America	90	NA	39.6	Company or office	Questionnaire	NECP ATP-III	<6 ≥6	aOR 2.30 (0.45−6.50) Ref.
Sabanayagam et al. (56)	Cross-sectional	USA, North America	4,307	44.6 ± 0.5 >20	49.9	Community	Standard questionnaire	AHA-NHLBI	<5 7 ≥9	aOR 1.24 (0.98-1.57) Ref. aOR 0.79 (0.69-1.36)
Wu et al. (57) (male)	Cross-sectional	Taiwan, Asia	2,772	44.9 ± 11.1	100	Hospital	Questionnaire	Modified NECP ATP-III	<6 7 >8	aOR 1.28 (1.01-1.63) Ref. aOR 1.43 (0.82-2.48)
Wu et al. (57) (female)	Cross-sectional	Taiwan, Asia	2,287	44.9 ± 11.1	0	Hospital	Questionnaire	Modified NECP ATP-III	<6 7 >8	aOR 1.04 (0.72-1.51) Ref. aOR 0.90 (0.32-2.51)
Hung et al. (58)	Cross-sectional	Taiwan, Asia	3,435	NA	64.4	Hospital	Standard questionnaire	AHA-NHLBI	<6 7-8 >8	aOR 1.02 (0.56-1.48) Ref. aOR 1.56 (1.38-1.74)
Yoo et al. (59)	Cross-sectional	USA, North America	96	42.4 ± 8.3	0	Company or office	Questionnaire	AHA-NHLBI	≤6 7-8 ≥8	aOR 2.30 (0.71-7.50) Ref. aOR 4.89 (1.32-18.13)
Okubo et al. (60)	Cross-sectional	Japan, Asia	1,481	57.5 ± 14.0	37.1	Community	Standard questionnaire	Japanese criteria 2005	<5 ≥6	aOR 1.20 (0.13-10.91) Ref.
Saleh and Janssen (61)	Cross-sectional	USA, North America	1,371	57.9 ± 13.6 ≥20	56.0	Community	Objective	AHA-NHLBI	<5 7.2-8.6 ≥9	aOR 0.91 (0.62-1.33) Ref. aOR 0.95 (0.66-1.39)
Yu et al. (62) (male)	Cross-sectional	China, Asia	1,618	54.4 ± 10.8 ≥35	100	Community	Questionnaire	Modified NECP ATP-III	≤7 7-8 >9	aOR 0.95 (0.83-1.10) Ref. aOR 1.17 (0.82-1.67)
Yu et al. (62) (female)	Cross-sectional	China, Asia	4,488	53.4 ± 10.3 ≥35	0	Community	Questionnaire	Modified NECP ATP-III	≤7 7-8 >9	aOR 0.99 (0.90-1.09) Ref. aOR 1.17 (0.92-1.49)
Canuto et al. (63)	Cross-sectional	Brazil, South America	902	31.0 ± 8.7	34.1	Company or office	Questionnaire	AHA-NHLBI	<5 ≥5	aOR 1.70 (1.09-2.24) Ref.
Chang et al. (64)	Cross-sectional	Taiwan, Asia	796	37.1 ± 7.6 20–60	100	Company or office	Standard questionnaire	AHA-NHLBI	<5 7-8 ≥8	aOR 1.04 (0.51-2.13) Ref. aOR 1.44 (0.69-2.98)
Wu et al. (65) (male)	Cross-sectional	China, Asia	11,380	63.6 ± 7.7	100	Company or office	Questionnaire	IDF	<7 7-8 ≥10	aOR 1.04 (0.93-1.17) Ref. aOR 1.01 (0.94-1.10)
Wu et al. (65) (female)	Cross-sectional	China, Asia	13,804	63.6 ± 7.7	0	Company or office	Questionnaire	IDF	<7 7-8 ≥10	aOR 0.93 (0.79-1.10) Ref. aOR 1.10 (0.97-1.24)
Lin et al. (66)	Cross-sectional	Taiwan, Asia	4,197	NA	46.0	Community	Questionnaire	IDF	<7 7-8 ≥9	aOR 1.54 (1.05-2.47) Ref. aOR 1.12 (0.70-1.82)
Min et al. (67)	Cross-sectional	Korea, Asia	8,558	20–75	0	Community	Questionnaire	NECP ATP-III	≤5 7 ≥9	aOR 0.75 (0.59-0.85) Ref. aOR 0.83 (0.68-1.02)
Xiao et al. (68) (male)	Cross-sectional	China, Asia	13,505	18–74	100	Community	Questionnaire	IDF	≤7 >8	Ref. aOR 1.20 (0.95-1.52)
Xiao et al. (68) (female)	Cross-sectional	China, Asia	6,977	18–74	0	Community	Questionnaire	IDF	≤7 >8	Ref. aOR 1.16 (1.00-1.35)
Cole et al. (69)	Cross-sectional	Chana, Africa	263	46.0 ± 11.6	41.0	Community	Objective	AHA-NHLBI	<7 7-8 >8	aOR 0.96 (0.39-1.38) Ref. aOR 1.98 (0.92-4.26)
Suliga et al. (70) (male)	Cross-sectional	Polish, Europe	3,056	37–66	100	Community	Questionnaire	NECP ATP-III	≤6 7-8 ≥9	aOR 0.99 (0.91-1.07) Ref. aOR 1.14 (0.97-1.35)
Suliga et al. (70) (female)	Cross-sectional	Polish, Europe	7,311	37–66	0	Community	Questionnaire	NECP ATP-III	≤6 7-8 ≥9	aOR 1.00 (0.94-1.07) Ref. aOR 1.05 (0.95-1.16)
Kaira et al. (71)	Cross-sectional	Dutch, Europe	1,679	60.8 ± 6.4 40–75	47.4	Community	Standard questionnaire	NECP ATP-III	<7 7-8 ≥9	aOR 0.96 (0.7-1.3) Ref. aOR 0.85 (0.6-1.2)
Kim et al. (72) (male)	Cross-sectional	Korea, Asia	44,930	40–69	100	Community	Interview	NECP ATP-III	<6 6-8 8-10	aOR 1.12 (1.05–1.19) Ref. aOR 1.01 (0.96-1.06)
Kim et al. (72) (female)	Cross-sectional	Korea, Asia	88,678	40–69	0	Community	Interview	NECP ATP-III	<6 6-8 8-10	aOR 1.05 (1.00-1.10) Ref, aOR 1.08 (1.04-1.12)
Ostadrahimi et al. (73)	Cross-sectional	Iran, Asia	14,916	35–70	50.0	Community	Standard questionnaire	NECP ATP-III	<6 6-9 >9	aOR 0.97 (0.85-1.10) Ref. aOR 1.18 (1.05-1.33)
Titova et al. (74)	Cross-sectional	Sweden, Europe	19,691	60.4 ± 8.5	44.0	Community	Interview	AHA-NHLBI	≤6 7-8 ≥9	aOR 1.08 (1.03-1.13) Ref. aOR 1.26 (1.14-1.39)
Qian et al. (75)	Cross-sectional	China, Asia	4,579	67.6 ± 6.3 >60	48.0	Community	Questionnaire	NECP ATP-III	<7 7-8 8-9	aOR 1.59 (1.10-2.33) Ref. aOR 1.42 (1.10-1.82)

Tables 2, 3 present the characteristics of all 36 studies. The individuals were all adults. The mean (SD) age of the individuals ranged from 31 (8.7) to 67.6 (7.3) years. The studies were conducted on five continents, 60% of which were performed in Asia. The definitions of short and long sleep durations varied between studies. Approximately 75% of studies defined a “short sleep duration” as < 6 or < 7 h, and ~80% of studies defined a “long sleep duration” as > 8 or > 9 h.

### Primary Analysis

#### Sleep Duration and the Risk of New-Onset Metabolic Syndrome

**Figure 2:**
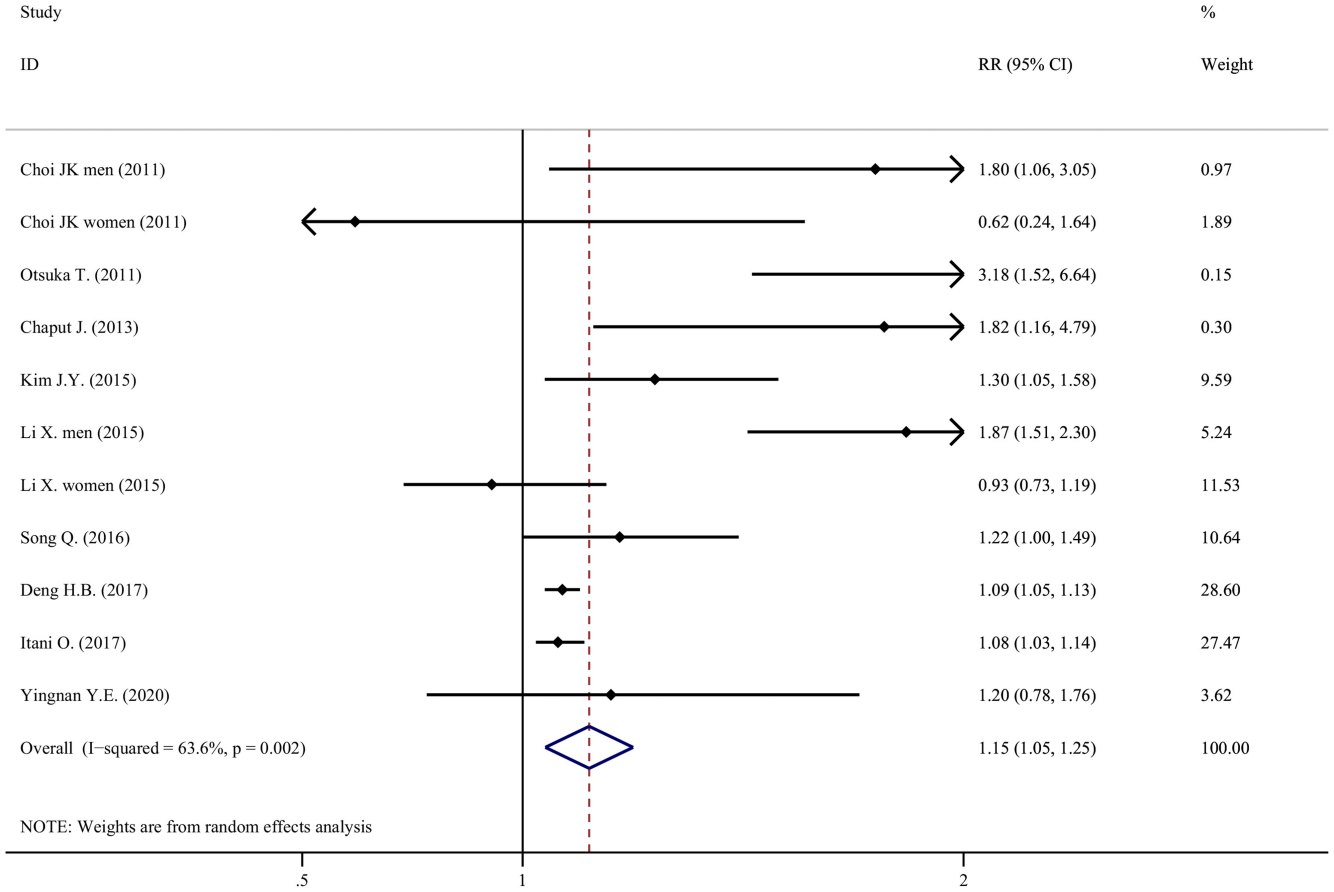
Forest plot of the association between short sleep duration and the risk of metabolic syndrome.

Compared with a moderate sleep duration, short sleep duration was associated with a statistically significant increase in new-onset metabolic syndrome, with an RR of 1.15 (95% CI = 1.05–1.25, *P* < 0.001, *I*^2^ = 63.6%, N of datasets = 11; Figure 2).

**Figure 3:**
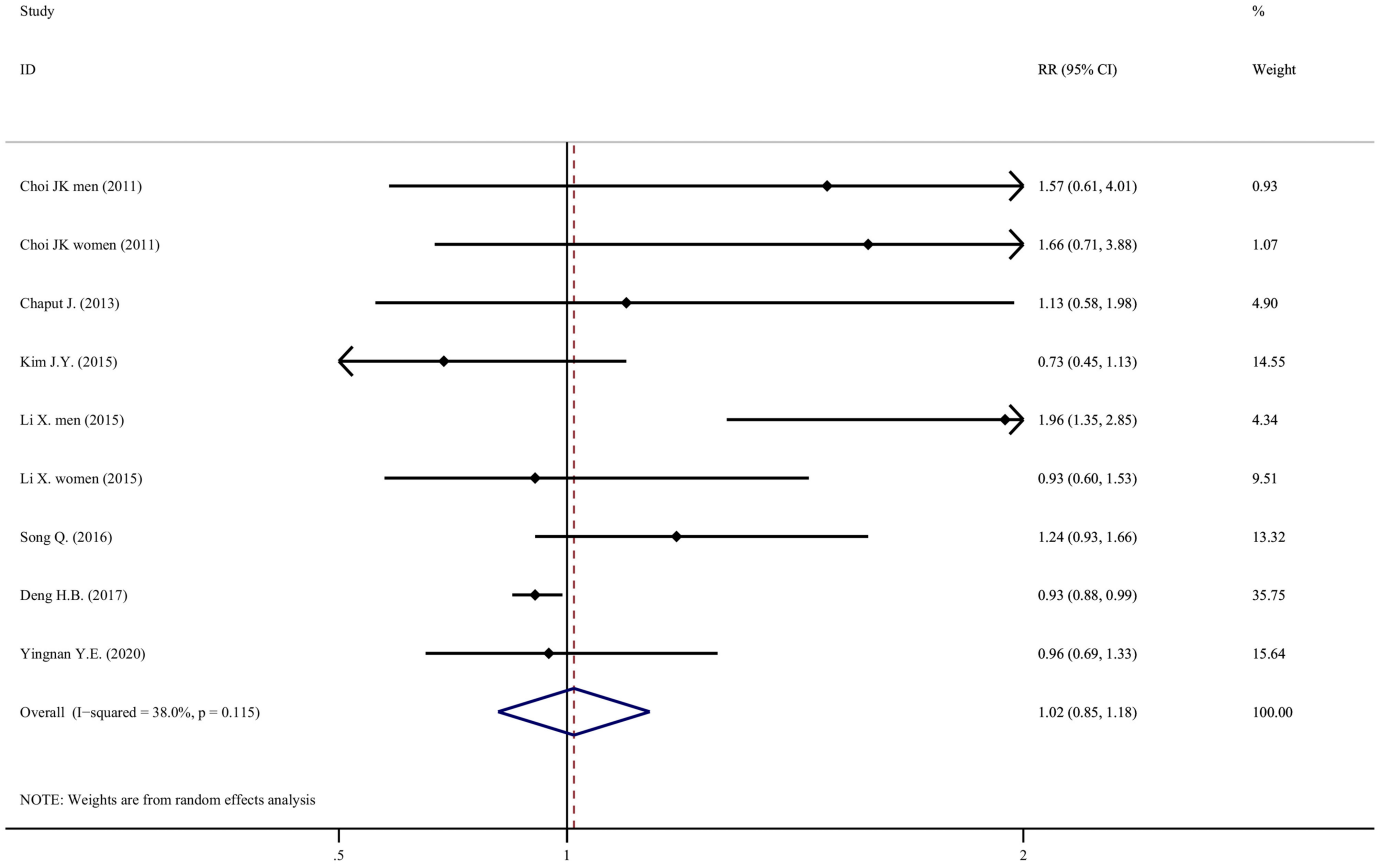
Forest plot of the association between long sleep duration and the risk of metabolic syndrome.

Compared with moderate sleep duration, the association between long sleep duration and the risk of metabolic syndrome was not statistically significant, with an RR of 1.02 (95% CI = 0.85–1.18, *P* = 0.491, *I*^2^ = 38.0%, *N* = 9; Figure 3), using a random-effect model. The RR was reduced to 0.94 (95% CI = 0.89–0.99, *P* = 0.050, *I*^2^ = 38.0%, *N* = 9; data not shown) using a fixed-effect model.

Among the seven studies that examined the effect of long sleep duration, six did not observe a significant association. Only Li X. found that a long sleep duration increased the risk of metabolic syndrome among men (adjusted HR = 1.96, 95% CI = 1.35–2.85).

#### Sleep Duration and the Prevalence of Metabolic Syndrome

**Figure 4:**
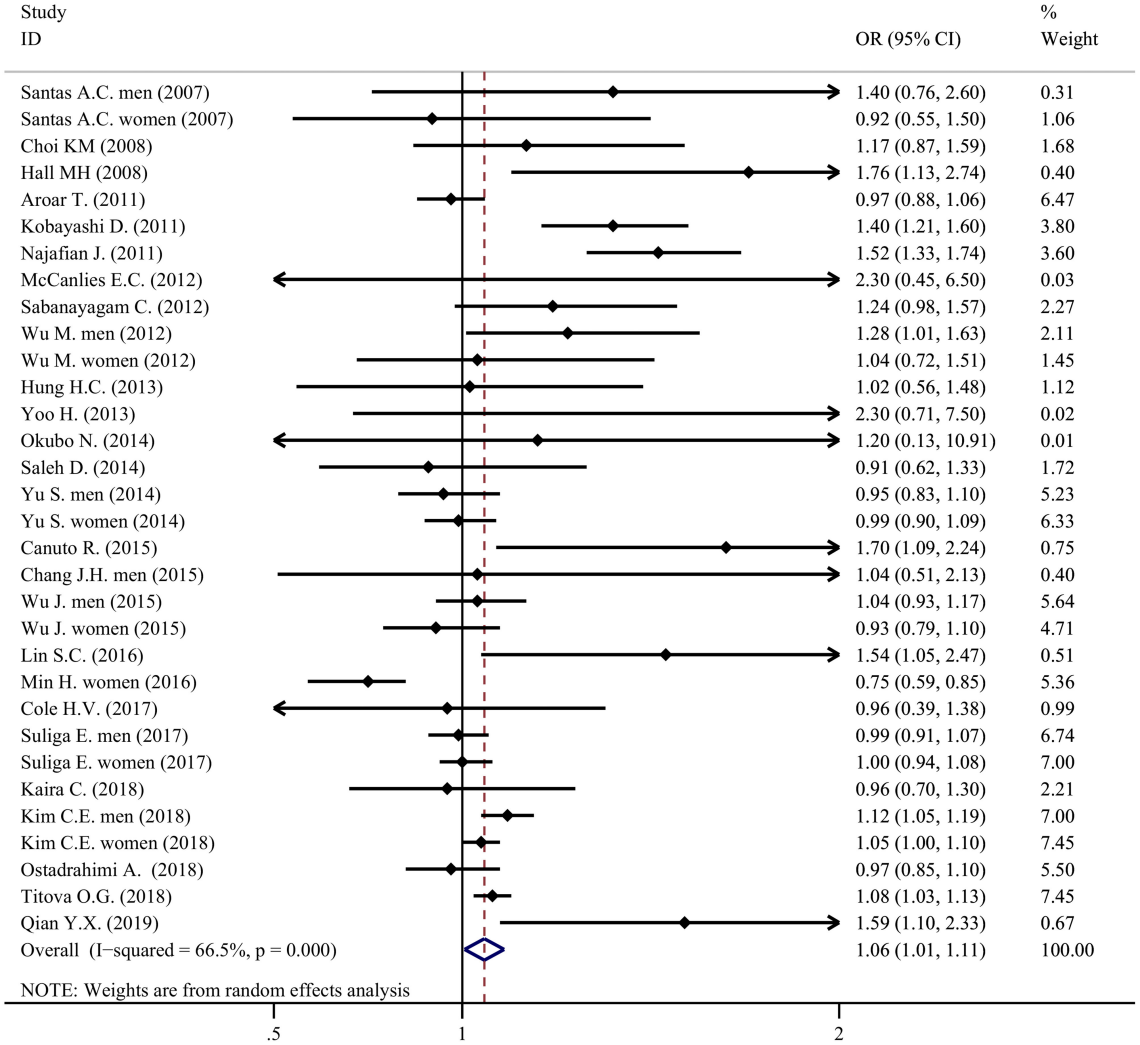
Forest plot of the association between short sleep duration and the prevalence of metabolic syndrome.

**Figure 5:**
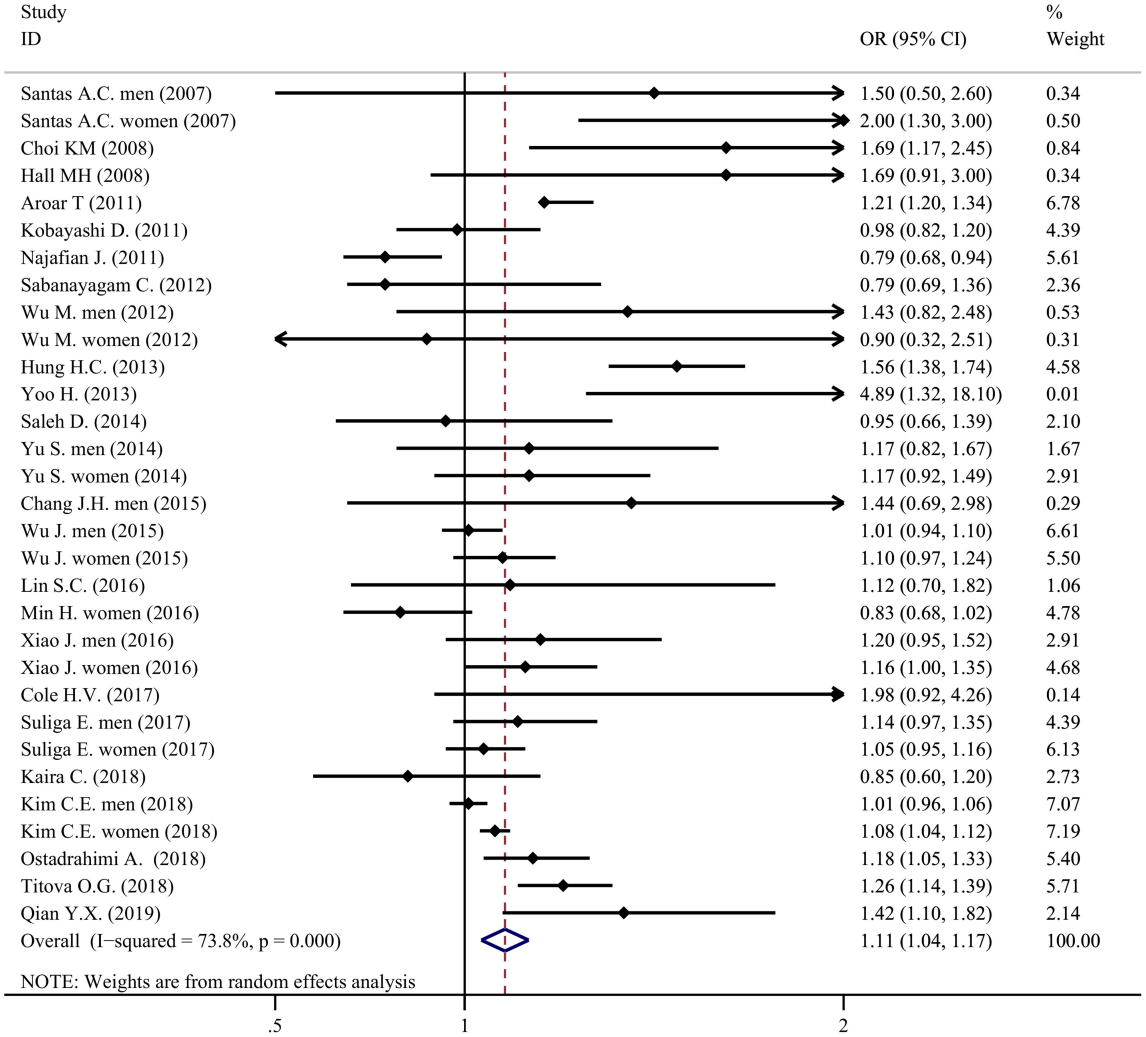
Forest plot of the association between long sleep duration and the prevalence of metabolic syndrome.

Compared with individuals with moderate sleep duration, people with a short or long sleep duration had a higher prevalence of metabolic syndrome. The pooled OR of metabolic syndrome in individuals with a short sleep duration compared to individuals with a moderate sleep duration was 1.06 (95% CI = 1.01–1.11, *P* < 0.001, *I*^2^ = 66.5%, *N* = 32; Figure 4). The pooled OR of metabolic syndrome in individuals with a long sleep duration compared to individuals with a moderate sleep duration was 1.11 (95% CI = 1.04-1.17, *P* < 0.001, *I*^2^ = 73.8%, *N* = 31; Figure 5).

### Possible Publication Bias in the Primary Analysis

The results of the Begg and Mazumdar and Egger tests are shown in Supplementary Table 2. No significant publication bias was observed. The “trim and fill” test indicated that the primary results remained significant after the data from the missing studies were filled (Supplementary Table 3). A visual inspection of the funnel plots also did not reveal apparent publication bias (Supplementary Figure 2).

### Subgroup Analysis of Cross-Sectional Studies

The results from the subgroup analysis of the cross-sectional studies were shown in Table 4. No significant difference was observed for subgroups stratified by gender or continent. For other subgroups, we identified statistically significant effects of the subgroup (*P* < 0.05 for heterogeneity between groups). For individuals with short sleep duration, the specific subgroups were the study population, sleep measurement, measures of metabolic syndrome, and sample size. For individuals with long sleep duration, the specific subgroups were sleep measurement, definition of long sleep duration, measures of metabolic syndrome, sample size, and study quality. However, there was still unexplained heterogeneity (*I*^2^ > 50%) within some subgroups. In conclusion, the subgroup couldn't fully explain the overall heterogeneity.

**Table 4:** Subgroup meta-analysis of cross-sectional studies.

	**Short sleep duration**				**Long sleep duration**			
	**No**.	**OR (95% CI)**	***I*^**2**^**	**${\text{P}}_{\text{z}}^{\text{a}}$**	**No**.	**OR (95% CI)**	***I*^**2**^**	**P_**z**_**
**Sex**								
Male	7	1.05 (0.98, 1.12)	46.0	Ref.	7	1.03 (0.99, 1.08)	14.0	Ref.
Female	7	0.99 (0.89, 1.09)	56.8	0.121	7	1.09 (1.00, 1.18)	67.9	0.272
		*P* = 0.174^b^				*P* = 0.104		
**Continent**								
Asia	19	1.08 (1.01, 1.17)	75.8	Ref.	20	1.12 (1.05, 1.20)	79.0	Ref.
Europe	6	1.03 (0.98, 1.08)	20.8	0.271	6	1.17 (1.01, 1.36)	64.6	0.583
North America	5	1.31 (0.99, 1.74)	24.5	0.205	4	1.22 (0.75, 2.00)	15.0	0.736
South America	1	1.70 (1.19, 2.44)	0.0	0.016	1	1.08 (0.92, 4.26)	0.0	0.147
Africa	1	0.96 (0.51, 1.81)	0.0	0.708		*P* = 0.171		
		*P* = 0.173						
**Study population**								
Community	21	1.03 (0.98, 1.08)	57.7	Ref.	22	1.14 (1.07, 1.21)	65.9	Ref.
Hospital	5	1.36 (1.21, 1.53)	45.7	<0.001	5	1.10 (0.77, 1.59)	91.6	0.885
Company or office	6	1.15 (0.93, 1.41)	37.6	0.334	4	1.09 (0.93, 1.28)	0.0	0.644
		*P* < 0.001				*P* = 0.261		
**Sleep measurement**								
Questionnaire	16	1.08 (0.99, 1.18)	69.4	Ref.	15	1.11 (1.03, 1.20)	87.6	Ref.
Interview	8	1.13 (1.04, 1.23)	76.3	0.496	8	1.12 (1.01, 1.24)	36.6	0.893
Standard questionnaire	6	1.02 (0.92, 1.13)	0.0	0.363	6	1.13 (0.90, 1.40)	81.1	0.947
Objective	2	0.92 (0.67, 1.28)	0.0	0.349	2	1.27 (0.63, 2.56)	28.3	0.733
		*P* = 0.012				*P* = 0.021		
**Definition of sleep duration**								
<5 h short or > 9 h long^c^	5	1.23 (0.98, 1.5)	29.2	Ref.	7	1.09 (0.92, 1.29)	67.3	Ref.
<6 h short or > 8 h long	13	1.13 (1.02, 1.24)	82.4	0.492	17	1.18 (1.09, 1.27)	53.6	0.405
<7 h short or > 7 h long	2	1.04 (0.98, 1.11)	20.4	0.164	6	1.01 (0.92, 1.11)	93.0	0.464
<8 h short	2	0.98 (0.90, 1.06)	0.0	0.065		*P* = 0.121		
		*P* = 0.154						
**MetS measurement**								
NECP ATP-III	12	1.06 (0.98, 1.16)	68.2	Ref.	10	1.07 (0.98, 1.15)	73.8	Ref.
Modified NECP ATP-III	6	1.00 (0.94, 1.07)	34.3	0.308	6	1.22 (1.15, 1.28)	35.8	0.006
AHA-NHLBI	9	1.20 (1.03, 1.39)	44.3	0.186	9	1.23 (1.01, 1.50)	27.3	0.195
IDF	3	1.05 (0.88, 1.25)	21.0	0.879	5	1.07 (1.01, 1.14)	33.4	0.945
Others	2	1.40 (1.22, 1.61)	14.1	<0.001	1	0.98 (0.81, 1.19)	10.1	0.426
		*P* < 0.001				*P* < 0.001		
**Sample size**								
<5,000	23	1.07 (0.99, 1.06)	49.7	Ref.	18	1.28 (1.12, 1.47)	56.2	Ref.
5,000–20,000	5	1.09 (0.96, 1.25)	57.4	0.842	8	1.05 (0.95, 1.15)	82.0	0.018
>20,000	4	1.11 (1.00, 1.23)	51.3	0.638	5	1.09 (1.01, 1.18)	82.1	0.044
		*P* = 0.004				*P* < 0.001		
**Study quality**								
High	7	1.03 (1.00, 1.06)	26.9	Ref.	7	1.03 (0.97, 1.09)	60.5	Ref.
Low	25	1.06 (1.03, 1.09)	63.4	0.013	23	1.15 (1.06, 1.25)	70.7	0.030
		*P* = 0.143				*P* < 0.001		

a *P-value of the two-sample z test for estimates between subgroups*.

b *P-value for heterogeneity*.

c *For effects of short sleep duration, this category included those who defined <5 h as short sleep duration. For effects of long sleep duration, this category included those who defined > 9 h as long sleep duration. The same meaning for the following terms*.

There was no significant difference between women and men with either short (*P* = 0.121) or long (*P* = 0.272) sleep durations. Sixty percent of studies were conducted in Asia. For a short sleep duration, the association was more evident for South America (OR = 1.70, 95% CI 1.19–2.44, *P* = 0.016, *N* = 1) than for Asia (OR = 1.08, 95% CI 1.01–1.17, *N* = 19). No detectable difference was identified between studies conducted in Asia and studies on other continents. For individuals with short sleep duration, hospital-based participants (OR = 1.36, 95% CI 1.21–1.53, *N* = 5) had a higher pooled OR than the community-based participants (OR = 1.03, 95% CI 0.98–1.08, *N* = 21). A significant difference was not observed between groups stratified by the methods used to measure the sleep duration (interview, standard questionnaire, and objective measurement in comparison with questionnaire). For a long sleep duration, studies using the Modified NECP ATP-III criteria (OR = 1.22, 95% CI 1.15–1.28, *N* = 6) had a higher overall OR value than studies using the NECP ATP-III criteria (OR = 1.07, 95% CI 0.98–1.15, *N* = 12). For a long sleep duration, the OR was lower in the studies with larger sample sizes. For both short and long sleep durations, the OR was lower in the studies of high quality.

### Sensitivity Analysis

None of the sensitivity analyses substantially altered the effects of both long and short sleep durations on metabolic syndrome (Supplementary Figure 1).

### Meta-Regression Analysis

A multivariable meta-regression analysis (Table 5) was conducted on cross-sectional studies to examine the potential effects of different factors on the natural logarithm of the OR of short or long sleep duration with the prevalence of MetS. For individuals with short sleep duration, a shorter definition of the duration was associated with a higher OR (*P* = 0.011 for the multivariable test and *P* = 0.099 for the univariable test). Higher study quality was associated with a lower OR (*P* = 0.010 for the univariable test and *P* = 0.033 for the multivariable test). The effect of the mean age was significant. However, the clinical effect (coef = -0.01) was limited. For individuals with long sleep duration, none of the study factors was significant.

**Table 5:** Results of the meta-regression analysis of cross-sectional studies.

	**Univariable analysis**		**Multivariable analysis**	
	**Coef**.	***P* value**	**Coef**.	***P* value**
	**Short sleep duration**			
Mean age, years	−0.01 (−0.02, −0.01)	0.020	−0.01 (−0.01, 0.00)	0.086
Proportion of males, %	0.08 (−0.10, 0.27)	0.356	−0.05 (−0.23, 0.14)	0.622
Definition of short/long sleep durations	−0.07 (−0.15, 0.01)	0.099	−0.08 (−0.15, −0.12)	0.011
Sample size	0.01 (−0.08, 0.09)	0.864	0.01 (−0.09, 0.09)	0.351
Study quality	−0.15 (−0.30, 0.00)	0.039	−0.26 (−0.61, 0.09)	0.149
	**Long sleep duration**			
Mean age, years	0.00 (0.00, 0.01)	0.113	0.00 (−0.00, 0.01)	0.720
Proportion of males, %	0.01 (−0.18, 0.02)	0.891	−0.04 (−0.44, 0.35)	0.828
Definition of short/long sleep durations	−0.16 (−0.33, 0.10)	0.776	−0.09 (−0.28, 0.10)	0.958
Sample size	−0.09 (−0.18, 0.00)	0.056	−0.08 (−0.25, 0.08)	0.318
Study quality	−0.01 (−0.14, 0.10)	0.807	0.01 (−0.50, 0.52)	0.360

## Discussion

To our knowledge, this meta-analysis is the most comprehensive study that has explored the relationship between sleep duration and metabolic syndrome. Currently, an increasing number of studies have linked both short and long sleep durations to adverse health outcomes (10, 26, 27). By combining the data from nine cohort studies, the present study showed that short sleep duration, instead of a long sleep duration, increased the risk of developing metabolic syndrome. In cross-sectional studies, both short and long sleep durations were associated with a higher prevalence of metabolic syndrome.

Our findings contribute important new information to previous reviews because of the separation of cohort studies and cross-sectional studies, our updated literature search, and the use of subgroup analysis. Three meta-analyses reported the association between sleep duration and metabolic syndrome. *Ju 2013* and *Iftikhar 2015* reported that only short sleep duration was associated with metabolic syndrome (11, 12), while *Xi 2014* identified associations of both short and long sleep durations to metabolic syndrome (13). Notably, *Ju 2013* pooled two cohort studies to examine the effect of short sleep duration on metabolic syndrome and only included one cohort study assessing the effect of long sleep duration on metabolic syndrome. One of the two cohort studies by *Otsuka 2011* (28) was of low quality because of its comparability.

We conducted a comprehensive subgroup analysis and meta-regression analysis of cross-sectional studies. Both our results and the results from previous studies showed no difference between sexes. The OR of studies conducted in Asia was not different from studies performed on other continents, except for South America. *Ju 2013* reported a difference between Asia and Europe. We attributed their findings to the limited number of included studies. Hospitalized patients with a short sleep duration had a higher prevalence of metabolic syndrome. Not surprisingly, hospital-based participants with worse health conditions more easily developed metabolic syndrome. Recently, an objective measurement of sleep duration has been considered more reliable than a subjective measurement. We did not observe a difference between the sleep duration recorded by questionnaire or objective measurement. Both a subgroup analysis and meta-regression analysis were used to examine the effects of the sample size and study quality on the pooled OR. Only a higher study quality was robustly associated with a lower OR for short sleep duration. In the multivariable meta-regression analysis, shorter sleep duration was linearly associated with a higher prevalence of metabolic syndrome. Longer sleep duration did not exhibit a linear association. This “J-shaped” association was quite different from the “U-shaped” association between sleep duration and health outcomes reported in many articles (29). However, this result should be interpreted cautiously, since “sleep duration” was a cut-off point defined by different studies examining different ethnicities in the meta-regression analysis. In one specific study, the author calculated the association among participants from the same ethnicity.

Several mechanisms linked sleep duration to metabolic syndrome. A short sleep duration might lead to the endocrine changes described below by affecting carbohydrate metabolism, the hypothalamo-pituitary-adrenal axis, and sympathetic activity. Decreased glucose tolerance and insulin sensitivity would increase glucose levels; increased levels of ghrelin, decreased levels of leptin, and increased appetite correlate with higher waist circumferences; and increased cortisol concentrations are associated with higher blood pressure (30, 31). Individuals with a short sleep duration tend to present elevated levels of high-sensitivity C-reactive protein and IL-6, which correlate with cardiovascular events (32, 33). A long sleep duration is linked to sleep fragmentation, which would cause numerous health outcomes, including metabolic changes (34). Individuals with a long sleep duration also have less time for excise, which might contribute to the association (35). Both short and long sleep durations display bidirectional associations with circadian rhythm, which is a risk factor for metabolic disorders (36, 37). Nonetheless, researchers have not yet clearly determined whether sleep duration is a causal risk factor for metabolic syndrome (38). Cohort studies are still unable to determine causality, although they have more power than cross-sectional studies. We must further examine the effect of changes in sleep duration (39) and perform a product Mendelian randomization study, a method using measured variation in genes, to prove a causal relationship.

The foremost strength of our study is that we pooled cohort studies and cross-sectional studies separately, which prevented misinterpretation of the results. By including nine cohort studies, we found that only a short sleep duration increased the incidence of metabolic syndrome. However, some limitations should be considered. First, most studies obtained the sleep duration using subjective measurements, such as interviews and questionnaires. Only two studies used objective measurements. We believe that subjective measurements would still be more applicable and utilized in epidemiological studies, although they have less accuracy and validity. Second, the cut-off points of short and long sleep duration and definition of metabolic syndrome varied between countries and studies (27). This limitation prevented us from translating our results into practical advice for the public. Third, we did not include other dimensions of sleep, such as sleep quality and sleep-disordered breathing. Sleep quality is a mechanism linking a short or long sleep duration with negative health outcomes (40). Fourth, we only included nine cohort studies, which prevented us from conducting further research, such as a subgroup analysis and meta-regression analysis.

## Conclusions

A short sleep duration, rather than a long sleep duration, was associated with a significant increase in the incidence of metabolic syndrome. Both short and long sleep durations were cross-sectionally associated with a high prevalence of metabolic syndrome. A sufficient sleep duration should be recommended to prevent metabolic syndrome.

## Data Availability Statement

The original contributions presented in the study are included in the article/Supplementary Material, further inquiries can be directed to the corresponding author/s.

## Author Contributions

HW and JH contributed to the conception and design of the study. JH and HJ organized the database and performed the statistical analyses. JH wrote the first draft of the manuscript. QF and HJ reviewed the manuscript. All authors approved the final version of the paper.

## Conflict of Interest

The authors declare that the research was conducted in the absence of any commercial or financial relationships that could be construed as a potential conflict of interest.

## References

[1] Engin A. The definition and prevalence of obesity and metabolic syndrome. Adv Exp Med Biol. (2017) 960:1–17. 10.1007/978-3-319-48382-5_1 28585193

[2] Falkner B Cossrow ND. Prevalence of metabolic syndrome and obesity-associated hypertension in the racial ethnic minorities of the United States. Curr Hypertens Rep. (2014) 16:449. 10.1007/s11906-014-0449-5 24819559 PMC4083846

[3] Mazloomzadeh S Rashidi Khazaghi Z Mousavinasab N. The prevalence of metabolic syndrome in Iran: a systematic review and meta-analysis. Iran J Public Health. (2018) 47:473–80. 29900131 PMC5996331

[4] Li R Li W Lun Z Zhang H Sun Z Kanu JS. Prevalence of metabolic syndrome in Mainland China: a meta-analysis of published studies. BMC Public Health. (2016) 16:296. 10.1186/s12889-016-2870-y 27039079 PMC4818385

[5] Isomaa B Almgren P Tuomi T Forsen B Lahti K Nissen M. Cardiovascular morbidity and mortality associated with the metabolic syndrome. Diabetes Care. (2001) 24:683–9. 10.2337/diacare.24.4.683 11315831

[6] Daly CA Hildebrandt P Bertrand M Ferrari R Remme W Simoons M. Adverse prognosis associated with the metabolic syndrome in established coronary artery disease: data from the EUROPA trial. Heart. (2007) 93:1406–11. 10.1136/hrt.2006.113084 17540689 PMC2016939

[7] Grundy SM. Drug therapy of the metabolic syndrome: minimizing the emerging crisis in polypharmacy. Nat Rev Drug Discov. (2006) 5:295–309. 10.1038/nrd2005 16582875

[8] Di Marzo V Silvestri C. Lifestyle and metabolic syndrome: contribution of the endocannabinoidome. Nutrients. (2019) 11:1956. 10.3390/nu11081956 31434293 PMC6722643

[9] Krittanawong C Tunhasiriwet A Wang Z Zhang H Farrell AM Chirapongsathorn S. Association between short and long sleep durations and cardiovascular outcomes: a systematic review and meta-analysis. Eur Heart J Acute Cardiovasc Care. (2019) 8:762–70. 10.1177/2048872617741733 29206050

[10] Jike M Itani O Watanabe N Buysse DJ Kaneita Y. Long sleep duration and health outcomes: a systematic review, meta-analysis and meta-regression. Sleep Med Rev. (2018) 39:25–36. 10.1016/j.smrv.2017.06.011 28890167

[11] Ju SY Choi WS. Sleep duration and metabolic syndrome in adult populations: a meta-analysis of observational studies. Nutr Diabetes. (2013) 3:e65. 10.1038/nutd.2013.8 23670223 PMC3671750

[12] Iftikhar IH Donley MA Mindel J Pleister A Soriano S Magalang UJ. Sleep duration and metabolic syndrome. an updated dose-risk metaanalysis. Ann Am Thorac Soc. (2015) 12:1364–72. 10.1513/AnnalsATS.201504-190OC 26168016 PMC5467093

[13] XI B. Short sleep duration predicts risk of metabolic syndrome: a systematic review and meta-analysis. Sleep Med Rev. (2014) 18:293–7. 10.1016/j.smrv.2013.06.001 23890470

[14] Tierney JF Stewart LA Ghersi D Burdett S Sydes MR. Practical methods for incorporating summary time-to-event data into meta-analysis. Trials. (2007) 8:16. 10.1186/1745-6215-8-16 17555582 PMC1920534

[15] Karen Grace-Martin AG. Cohort and Case-Control Studies: Pro's and Con's (2017). Available online at: https://www.theanalysisfactor.com/cohort-and-case-control-studies-pros-and-cons/

[16] Setia MS. Methodology series module 3: cross-sectional studies. Indian J Dermatol. (2016) 61:261–4. 10.4103/0019-5154.182410 27293245 PMC4885177

[17] Grant RL. Converting an odds ratio to a range of plausible relative risks for better communication of research findings. BMJ. (2014) 348:f7450. 10.1136/bmj.f7450 24464277

[18] Adenekan B Pandey A McKenzie S Zizi F Casimir GJ Jean-Louis G. Sleep in America: role of racial/ethnic differences. Sleep Med Rev. (2013) 17:255–62. 10.1016/j.smrv.2012.07.002 23348004 PMC3644542

[19] Wells GSB O'Connell D Peterson J Welch V Losos M. The Newcastle-Ottawa Scale (NOS) for assessing the quality of nonrandomised studies in meta-analyses (2014). Available online at: http://www.ohri.ca/programs/clinical_epidemiology/oxford.asp

[20] Higgins JP Thompson SG Deeks JJ Altman DG. Measuring inconsistency in meta-analyses. BMJ. (2003) 327:557–60. 10.1136/bmj.327.7414.557 12958120 PMC192859

[21] Egger M Davey Smith G Schneider M Minder C. Bias in meta-analysis detected by a simple, graphical test. BMJ. (1997) 315:629–34. 10.1136/bmj.315.7109.629 9310563 PMC2127453

[22] Begg CB Mazumdar M. Operating characteristics of a rank correlation test for publication bias. Biometrics. (1994) 50:1088–101. 10.2307/2533446 7786990

[23] Duval S Tweedie R. Trim and fill: a simple funnel-plot-based method of testing and adjusting for publication bias in meta-analysis. Biometrics. (2000) 56:455–63. 10.1111/j.0006-341X.2000.00455.x 10877304

[24] W. V. Conducting meta-analyses in R with the metafor package. J Stat Softw. (2010) 36:1–48. 10.18637/jss.v036.i03

[25] Zhang Tiansong ZS. How to compare summary estimates of different subgroups in meta-analysis. Chin J Evidence-Based Med. (2017). Available online at: https://www.ixueshu.com/document/be6c862ae782c141182f500f64e4272a318947a18e7f9386.html

[26] Itani O Jike M Watanabe N Kaneita Y. Short sleep duration and health outcomes: a systematic review, meta-analysis, and meta-regression. Sleep Med. (2017) 32:246–56. 10.1016/j.sleep.2016.08.006 27743803

[27] Hua J Sun H Shen Y. Improvement in sleep duration was associated with higher cognitive function: a new association. Aging (Albany NY). (2020) 12:20623–44. 10.18632/aging.103948 33082298 PMC7655193

[28] Otsuka T Kawada T Yanai M Kitagawa Y Kan H. [Incidence of metabolic syndrome and associated lifestyle factors in a worksite male population]. Sangyo Eiseigaku Zasshi. (2011) 53:78–86. 10.1539/sangyoeisei.B10013 21372516

[29] Smiley A King D Bidulescu A. The association between sleep duration and metabolic syndrome: the NHANES 2013/2014. Nutrients. (2019) 11:2582. 10.3390/nu11112582 31717770 PMC6893635

[30] Spiegel K Leproult R Van Cauter E. Impact of sleep debt on metabolic and endocrine function. Lancet. (1999) 354:1435–9. 10.1016/S0140-6736(99)01376-8 10543671

[31] Zimberg IZ Damaso A Del Re M Carneiro AM de Sa Souza H de Lira FS. Short sleep duration and obesity: mechanisms and future perspectives. Cell Biochem Funct. (2012) 30:524–9. 10.1002/cbf.2832 22473743

[32] Meier-Ewert HK Ridker PM Rifai N Regan MM Price NJ Dinges DF. Effect of sleep loss on C-reactive protein, an inflammatory marker of cardiovascular risk. J Am Coll Cardiol. (2004) 43:678–83. 10.1016/j.jacc.2003.07.050 14975482

[33] Hua J Qiao Y Ke C Shen Y. Higher visit-to-visit total cholesterol variability is associated with lower cognitive function among middle-aged and elderly Chinese men. Sci Rep. (2020) 10:15555. 10.1038/s41598-020-72601-7 32968174 PMC7511393

[34] Grandner MA Drummond SP. Who are the long sleepers? Towards an understanding of the mortality relationship. Sleep Med Rev. (2007) 11:341–60. 10.1016/j.smrv.2007.03.010 17625932 PMC3755488

[35] Stranges S Dorn JM Shipley MJ Kandala NB Trevisan M Miller MA. Correlates of short and long sleep duration: a cross-cultural comparison between the United Kingdom and the United States: the Whitehall II Study and the Western New York Health Study. Am J Epidemiol. (2008) 168:1353–64. 10.1093/aje/kwn337 18945686 PMC2727192

[36] Allebrandt KV Teder-Laving M Akyol M Pichler I Muller-Myhsok B Pramstaller P. CLOCK gene variants associate with sleep duration in two independent populations. Biol Psychiatry. (2010) 67:1040–7. 10.1016/j.biopsych.2009.12.026 20149345

[37] Lemmer B Oster H. The role of circadian rhythms in the hypertension of diabetes mellitus and the metabolic syndrome. Curr Hypertens Rep. (2018) 20:43. 10.1007/s11906-018-0843-5 29730779

[38] Kraemer HC Kazdin AE Offord DR Kessler RC Jensen PS Kupfer DJ. Coming to terms with the terms of risk. Arch Gen Psychiatry. (1997) 54:337–43. 10.1001/archpsyc.1997.01830160065009 9107150

[39] Buysse DJ. Sleep health: can we define it? Does it matter? Sleep. (2014) 37:9–17. 10.5665/sleep.3298 24470692 PMC3902880

[40] Musiek ES Holtzman DM. Mechanisms linking circadian clocks, sleep, and neurodegeneration. Science. (2016) 354:1004–8. 10.1126/science.aah4968 27885006 PMC5219881

[41] Choi JK Kim MY Kim JK Park JK Oh SS Koh SB. Association between short sleep duration and high incidence of metabolic syndrome in midlife women. Tohoku J Exp Med. (2011) 225:187–93. 10.1620/tjem.225.187 22001675

[42] Chaput JP McNeil J Despres JP Bouchard C Tremblay A. Short sleep duration as a risk factor for the development of the metabolic syndrome in adults. Prev Med. (2013) 57:872–7. 10.1016/j.ypmed.2013.09.022 24099879

[43] Kim JY Yadav D Ahn SV Koh SB Park JT Yoon J. A prospective study of total sleep duration and incident metabolic syndrome: the ARIRANG study. Sleep Med. (2015) 16:1511–5. 10.1016/j.sleep.2015.06.024 26611949

[44] Li X Lin L Lv L Pang X Du S Zhang W. U-shaped relationships between sleep duration and metabolic syndrome and metabolic syndrome components in males: a prospective cohort study. Sleep Med. (2015) 16:949–54. 10.1016/j.sleep.2015.03.024 26116460

[45] Song Q Liu X Zhou W Wang X Wu S. Changes in sleep duration and risk of metabolic syndrome: the Kailuan prospective study. Sci Rep. (2016) 6:36861. 10.1038/srep36861 27857185 PMC5114677

[46] Deng HB Tam T Zee BC Chung RY Su X Jin L. Short sleep duration increases metabolic impact in healthy adults: a population-based cohort study. Sleep. (2017) 40. 10.1093/sleep/zsx130. [Epub ahead of print]. 28977563

[47] Itani O Kaneita Y Tokiya M Jike M Murata A Nakagome S. Short sleep duration, shift work, and actual days taken off work are predictive life-style risk factors for new-onset metabolic syndrome: a seven-year cohort study of 40,000 male workers. Sleep Med. (2017) 39:87–94. 10.1016/j.sleep.2017.07.027 29157594

[48] Ye Y Zhang L Wang A Wang Y Wang S Ning G. Association of sleep duration with stroke, myocardial infarction, and tumors in a Chinese population with metabolic syndrome: a retrospective study. Lipids Health Dis. (2020) 19:155. 10.1186/s12944-020-01328-1 32593309 PMC7321539

[49] Santos AC Ebrahim S Barros H. Alcohol intake, smoking, sleeping hours, physical activity and the metabolic syndrome. Prev Med. (2007) 44:328–34. 10.1016/j.ypmed.2006.11.016 17239432

[50] Choi KM Lee JS Park HS Baik SH Choi DS Kim SM. Relationship between sleep duration and the metabolic syndrome: Korean National Health and Nutrition Survey 2001. Int J Obes (Lond). (2008) 32:1091–7. 10.1038/ijo.2008.62 18475274

[51] Hall MH Muldoon MF Jennings JR Buysse DJ Flory JD Manuck SB. Self-reported sleep duration is associated with the metabolic syndrome in midlife adults. Sleep. (2008) 31:635–43. 10.1093/sleep/31.5.635 18517034 PMC2398755

[52] Arora T Jiang CQ Thomas GN Lam KB Zhang WS Cheng KK. Self-reported long total sleep duration is associated with metabolic syndrome: the Guangzhou Biobank Cohort Study. Diabetes Care. (2011) 34:2317–9. 10.2337/dc11-0647 21873559 PMC3177714

[53] Kobayashi D Takahashi O Deshpande GA Shimbo T Fukui T. Relation between metabolic syndrome and sleep duration in Japan: a large scale cross-sectional study. Intern Med. (2011) 50:103–7. 10.2169/internalmedicine.50.4317 21245632

[54] Najafian J Toghianifar N Mohammadifard N Nouri F. Association between sleep duration and metabolic syndrome in a population-based study: Isfahan Healthy Heart Program. J Res Med Sci. (2011) 16:801–6. 22091310 PMC3214399

[55] McCanlies EC Slaven JE Smith LM Andrew ME Charles LE Burchfiel CM. Metabolic syndrome and sleep duration in police officers. Work. (2012) 43:133–9. 10.3233/WOR-2012-1399 22927620

[56] Sabanayagam C Zhang R Shankar A. Markers of sleep-disordered breathing and metabolic syndrome in a multiethnic sample of US adults: results from the National Health and Nutrition Examination Survey 2005–2008. Cardiol Res Pract. (2012) 2012:630802. 10.1155/2012/630802 22577590 PMC3347463

[57] Wu MC Yang YC Wu JS Wang RH Lu FH Chang CJ. Short sleep duration associated with a higher prevalence of metabolic syndrome in an apparently healthy population. Prev Med. (2012) 55:305–9. 10.1016/j.ypmed.2012.07.013 22846501

[58] Hung HC Yang YC Ou HY Wu JS Lu FH Chang CJ. The association between self-reported sleep quality and metabolic syndrome. PLoS ONE. (2013) 8:e54304. 10.1371/journal.pone.0054304 23342127 PMC3544823

[59] Yoo H Franke WD. Sleep habits, mental health, and the metabolic syndrome in law enforcement officers. J Occup Environ Med. (2013) 55:99–103. 10.1097/JOM.0b013e31826e294c 23207742

[60] Okubo N Matsuzaka M Takahashi I Sawada K Sato S Akimoto N. Relationship between self-reported sleep quality and metabolic syndrome in general population. BMC Public Health. (2014) 14:562. 10.1186/1471-2458-14-562 24903537 PMC4087247

[61] Saleh D Janssen I. Interrelationships among sedentary time, sleep duration, and the metabolic syndrome in adults. BMC Public Health. (2014) 14:666. 10.1186/1471-2458-14-666 24975509 PMC4086271

[62] Yu S Guo X Yang H Zheng L Sun Y. An update on the prevalence of metabolic syndrome and its associated factors in rural northeast China. BMC Public Health. (2014) 14:877. 10.1186/1471-2458-14-877 25159694 PMC4153886

[63] Canuto R Pattussi MP Macagnan JB Henn RL Olinto MT. Metabolic syndrome in fixed-shift workers. Rev Saude Publica. (2015) 49:30. 10.1590/S0034-8910.2015049005524 26061455 PMC4544368

[64] Chang JH Huang PT Lin YK Lin CE Lin CM Shieh YH. Association between sleep duration and sleep quality, and metabolic syndrome in Taiwanese police officers. Int J Occup Med Environ Health. (2015) 28:1011–23. 10.13075/ijomeh.1896.00359 26294202

[65] Wu J Xu G Shen L Zhang Y Song L Yang S. Daily sleep duration and risk of metabolic syndrome among middle-aged and older Chinese adults: cross-sectional evidence from the Dongfeng-Tongji cohort study. BMC Public Health. (2015) 15:178. 10.1186/s12889-015-1521-z 25885456 PMC4349607

[66] Lin SC Sun CA You SL Hwang LC Liang CY Yang T. The link of self-reported insomnia symptoms and sleep duration with metabolic syndrome: a Chinese population-based Study. Sleep. (2016) 39:1261–6. 10.5665/sleep.5848 27070137 PMC4863215

[67] Min H Um YJ Jang BS Shin D Choi E Park SM. Association between sleep duration and measurable cardiometabolic risk factors in Healthy Korean Women: the Fourth and Fifth Korean National Health and Nutrition Examination Surveys (KNHANES IV and V). Int J Endocrinol. (2016) 2016:3784210. 10.1155/2016/3784210 27956898 PMC5124459

[68] Xiao J Shen C Chu MJ Gao YX Xu GF Huang JP. Physical activity and sedentary behavior associated with components of metabolic syndrome among people in rural China. PLoS ONE. (2016) 11:e0147062. 10.1371/journal.pone.0147062 26789723 PMC4720370

[69] Cole HV Owusu-Dabo E Iwelunmor J Newsome V Meeks K Agyemang C. Sleep duration is associated with increased risk for cardiovascular outcomes: a pilot study in a sample of community dwelling adults in Ghana. Sleep Med. (2017) 34:118–25. 10.1016/j.sleep.2017.03.008 28522079

[70] Suliga E Koziel D Ciesla E Rebak D Gluszek S. Sleep duration and the risk of metabolic syndrome-A cross-sectional study. medRxiv. (2017). 10.5114/ms.2017.70342 27842207

[71] van der Pal KC Koopman ADM Lakerveld J van der Heijden AA Elders PJ Beulens JW. The association between multiple sleep-related characteristics and the metabolic syndrome in the general population: the New Hoorn study. Sleep Med. (2018) 52:51–7. 10.1016/j.sleep.2018.07.022 30278295

[72] Kim CE Shin S Lee HW Lim J Lee JK Shin A. Association between sleep duration and metabolic syndrome: a cross-sectional study. BMC Public Health. (2018) 18:720. 10.1186/s12889-018-5557-8 29895272 PMC5998453

[73] Ostadrahimi A Nikniaz Z Faramarzi E Mohammadpoorasl A Ansarin K Somi MH. Does long sleep duration increase risk of metabolic syndrome in Azar cohort study population? Health Promot Perspect. (2018) 8:290–5. 10.15171/hpp.2018.41 30479983 PMC6249496

[74] Titova OE Lindberg E Elmstahl S Lind L Schioth HB Benedict C. Associations between the prevalence of metabolic syndrome and sleep parameters vary by age. Front Endocrinol (Lausanne). (2018) 9:234. 10.3389/fendo.2018.00234 29867766 PMC5958301

[75] Qian YX Liu JH Ma QH Sun HP Xu Y Pan CW. Associations of sleep durations and sleep-related parameters with metabolic syndrome among older Chinese adults. Endocrine. (2019) 66:240–8. 10.1007/s12020-019-02064-y 31473919

[76] Moher D Liberati A Tetzlaff J Altman DG The PRISMA Group. Preferred reporting items for systematic reviews and meta-analyses: the PRISMA statement. PLoS Med. (2009) 6:e1000097. 10.1371/journal.pmed1000097 19621072 PMC2707599

