# Supplementary figures and images for: Sleep Duration and the Risk of Metabolic Syndrome in Adults: A Systematic Review and Meta-Analysis
Source: Front Neurol. 2021 Feb 18;12:635564. doi: 10.3389/fneur.2021.635564 (PMC7935510; doi:10.3389/fneur.2021.635564)

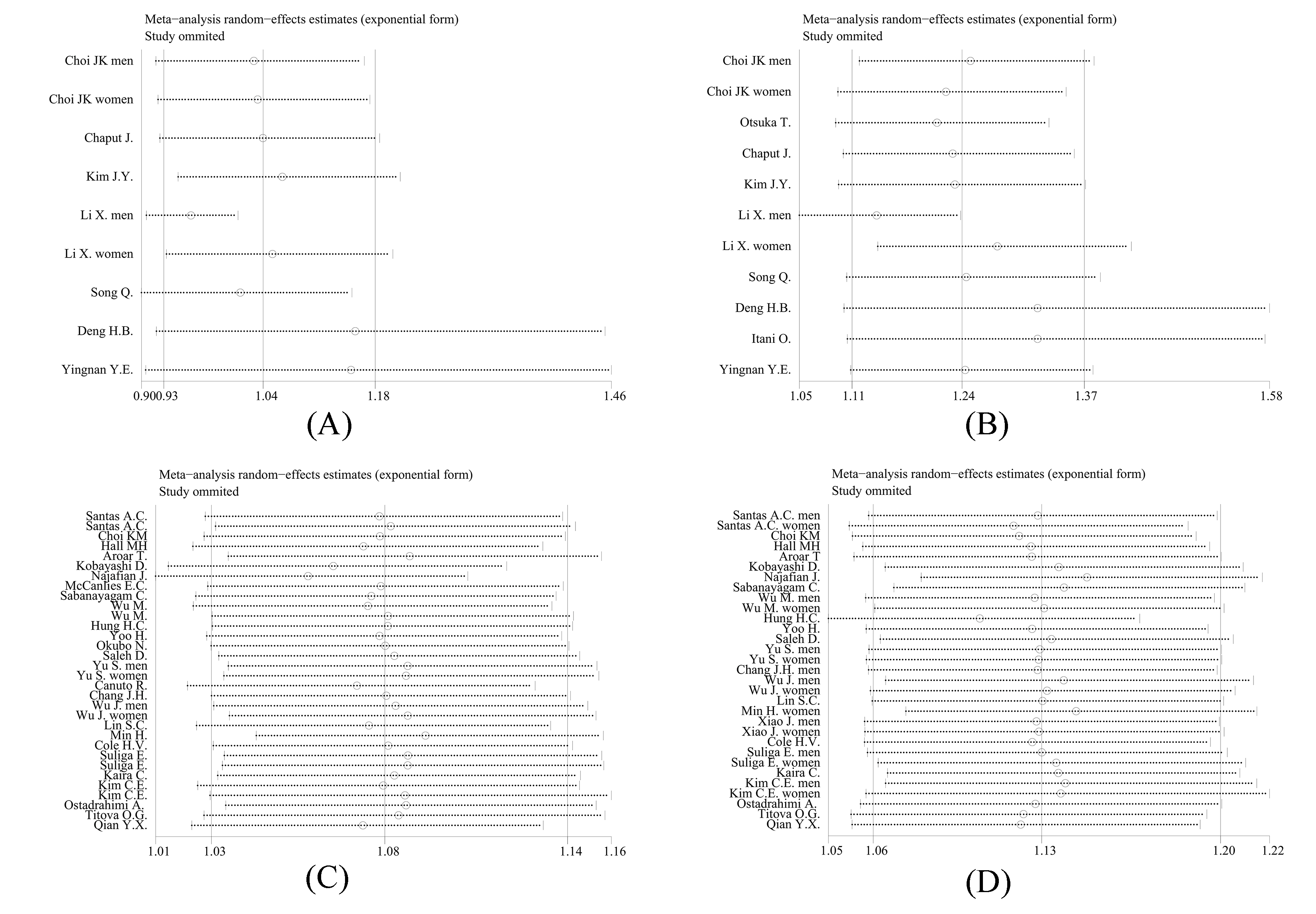

Supplement: Supplementary file 1 [file Image_1.TIF]

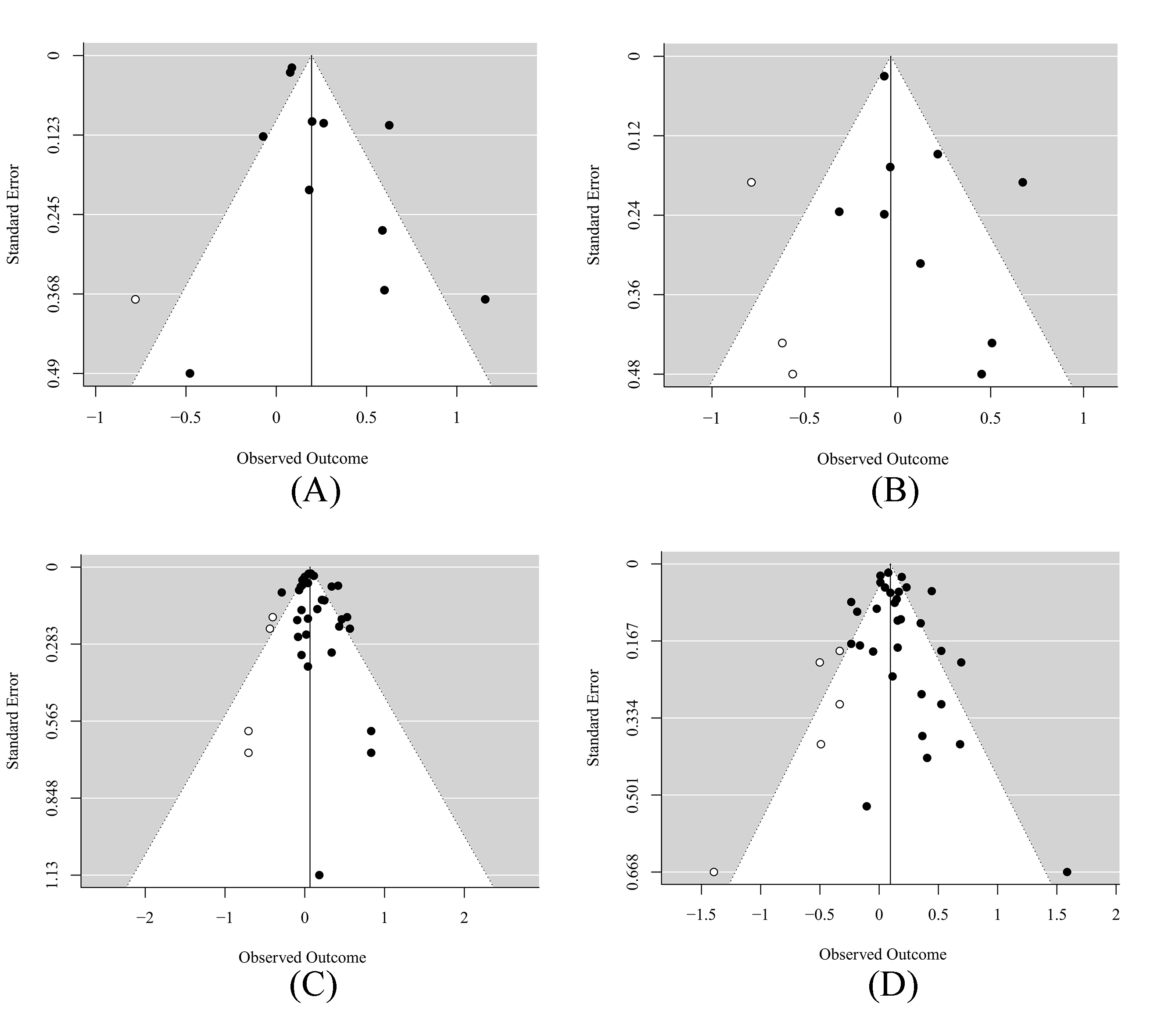

Supplement: Supplementary file 2 [file Image_2.TIF]
