# Supplementary material for: Sleep Duration and the Risk of Metabolic Syndrome in Adults: A Systematic Review and Meta-Analysis
Source: Front Neurol. 2021 Feb 18;12:635564. doi: 10.3389/fneur.2021.635564 (PMC7935510; doi:10.3389/fneur.2021.635564)
Supplement: Supplementary file 3 [file Data_Sheet_1.docx]

Supplementary Materials

**Jianian Hua^13,#^, Hezi Jiang^23,#^, Hui Wang^1,^* and Qi Fang^1,^****

^1^ Department of Neurology, The First Affiliated Hospital of Soochow University, Suzhou, 215006, Jiangsu Province, China.

^2^ Department of Cardiology, The First Affiliated Hospital of Soochow University, Suzhou, China.

^3^ Medical College of Soochow University, Suzhou, 215123, PR China.

^#^These authors contributed equally.

*** Correspondence:**

Hui Wang

dr_wanghui@sina.com

******** Correspondence:**
Qi Fang
fangqi_008@126.com

# List of Captions

| Captions | Contents |
| --- | --- |
| Table S1 | Quality appraisal using the modified Ottawa-Newcastle Scale |
| Table S2 | Publication bias |
| Table S3 | Results of “trim and fill” |
| Figure S1  Figure S2 | Sensitivity analyses  Funnel plots |
| Appendix S1 | Search terms |

# Tables

## Table S1. Quality appraisal using the modified Ottawa-Newcastle Scale

| Author, year | Design | Ottawa-Newcastle Scale | | |
| --- | --- | --- | --- | --- |
|  |  | Selection | Comparability | Exposure/outcome |
| Choi, 2011  (male) | Prospective cohort | ★★★★ | ★★ | ★★★ |
| Choi, 2011  (female) | PC | ★★★★ | ★★ | ★★★ |
| Otsuka, 2011 | PC | ★★★ | ★ | ★★★ |
| Chaput, 2013 | PC | ★★★ | ★ | ★★★ |
| Kim, 2015 | PC | ★★★ | ★★ | ★★ |
| Li, 2015  (male) | PC | ★★★★ | ★★ | ★★★ |
| Li, 2015  (female) | PC | ★★★★ | ★★ | ★★★ |
| Song, 2016 | PC | ★★★ | ★★ | ★★ |
| Deng, 2017 | PC | ★★★★ | ★★ | ★★★ |
| Itani, 2017 | PC | ★★★★ | ★ | ★★★ |
| Yingnan, 2020 | PC | ★★★ | ★★ | ★★★ |
|  |  | Modiefied Ottawa-Newcastle Scale | | |
| Santus, 2007  (male) | C-S | ★★★ | ★★ | ★★ |
| Santus, 2007  (female) | C-S | ★★★ | ★★ | ★★ |
| Choi, 2008 | C-S | ★★★ | ★★ | ★★ |
| Hall, 2008 | C-S | ★★★ | ★★ | ★★ |
| Aroar, 2011 | C-S | ★★★ | ★★ | ★★ |
| Kobayashi, 2011 | C-S | ★★ | ★★ | ★★ |
| Najafian, 2011 | C-S | ★★ | ★ | ★★ |
| McCanlies, 2012 | C-S | ★★ | ★ | ★★ |
| Sabanayagam, 2012 | C-S | ★★★ | ★★ | ★★ |
| Wu, 2012  (male) | C-S | ★★★ | ★★ | ★★ |
| Wu, 2012  (female) | C-S | ★★★ | ★★ | ★★ |
| Hung, 2013 | C-S | ★★ | ★★ | ★★ |
| Yoo, 2013 | C-S | ★★ | ★★ | ★★ |
| Okubo, 2014 | C-S | ★★★ | ★ | ★★ |
| Saleh, 2014 | C-S | ★★★ | ★★ | ★★★ |
| Yu, 2014  (male) | C-S | ★★★ | ★★ | ★★ |
| Yu, 2014  (female) | C-S | ★★★ | ★★ | ★★ |

Table S1 - Continued

| Author, year | Design | Modiefied Ottawa-Newcastle Scale | | |
| --- | --- | --- | --- | --- |
|  |  | Selection | Comparability | Exposure/outcome |
| Canuto, 2015 | C-S | ★★ | ★ | ★★ |
| Chang, 2015 | C-S | ★★★ | ★★ | ★★ |
| Wu, 2015  (male) | C-S | ★★★ | ★★ | ★★ |
| Wu, 2015  (female) | C-S | ★★★ | ★★ | ★★ |
| Lin, 2016 | C-S | ★★★ | ★★ | ★★ |
| Min, 2016 | C-S | ★★★★ | ★★ | ★★ |
| Xiao, 2016  (male) | C-S | ★★★ | ★ | ★★ |
| Xiao, 2016  (female) | C-S | ★★★ | ★ | ★★ |
| Cole,2017 | C-S | ★★★ | ★★ | ★★★ |
| Suliga, 2017  (male) | C-S | ★★★★ | ★★ | ★★ |
| Suliga, 2017  (female) | C-S | ★★★★ | ★★ | ★★ |
| Kaira, 2018 | C-S | ★★★ | ★★ | ★★ |
| Kim, 2018  (male) | C-S | ★★★★ | ★★ | ★★ |
| Kim, 2018  (female) | C-S | ★★★★ | ★★ | ★★ |
| Ostadrahimi, 2018 | C-S | ★★★ | ★★ | ★★ |
| Titova, 2018 | C-S | ★★★ | ★★ | ★★ |
| Qian, 2019 | C-S | ★★★ | ★★ | ★★ |

Abbreviations: PC, prospective cohort study; C-S, cross-sectional study.

## Table S2. Publication bias

|  | P for Egger test | P for Begg test |
| --- | --- | --- |
| Short, cohort | 0.077 | 0.312 |
| Long, cohort | 0.213 | 0.140 |
| Short, cross-sectional | 0.373 | 0.345 |
| Long, cross-sectional | 0.219 | 0.153 |

## Table S3. Results of “trim and fill”

|  | Corrected estimate^~~a~~^ | Original estimate |
| --- | --- | --- |
| Short, cohort | 1.22 (1.15, 1.29) | 1.15 (1.05, 1.15) |
| Long, cohort | 0.96 (0.86, 1.07) | 1.02 (0.85, 1.18) |
| Short, cross-sectional | 1.07 (1.04, 1.10) | 1.12 (1.08, 1.18) |
| Long, cross-sectional | 1.10 (1.07, 1.13) | 1.11 (1.04, 1.17) |

^~~a~~^The effect estimate after “trim and fill” correction.

# Figure legends

Figure S1. Sensitive analyses. Sensitivity analyses of association between sleep duration and metabolic syndrome by omitting one study each time and rerunning the analyses. (A) short sleep duration among cohort studies; (B) long sleep duration among cohort studies; (C) short sleep duration among cross-sectional studies; (D) long sleep duration among cross-sectional studies.

**Figure S2. Funnel plots.** Funnel plots for the metabolic syndrome. (A) short sleep duration among cohort studies; (B) long sleep duration among cohort studies; (C) short sleep duration among cross-sectional studies; (D) long sleep duration among cross-sectional studies.

# Appendix

## Appendix S1. Search terms.

**Search terms for MEDLINE through Pubmed (search data:May 1, 2020)**

("sleep duration"[All Fields] OR “sleep hour"[All Fields] OR “sleeping hour"[All Fields] OR "hours of sleep”[All Fields] OR "sleep time"[All Fields] OR "sleep length"[All Fields] OR "sleep period” [All Fields] OR “sleeping time”[All Fields]) AND (“metabolic syndrome”[All Fields] OR “MetS” [All Fields] OR “MS” [All Fields] OR “syndrome X” [All Fields] OR “cardiometabolic risk factor” [All Fields] OR “insulin resistance syndrome” [All Fields])

**Search terms for EMBASE through Ovid (search data:May 1, 2020)**

1. sleep duration.ti,ab,kw
2. sleep hour.ti,ab,kw
3. sleeping hour.ti,ab,kw
4. hours of sleep.ti,ab,kw
5. sleep time.ti,ab,kw
6. sleep length.ti,ab,kw
7. sleep period.ti,ab,kw
8. sleeping time.ti,ab,kw
9. sleep span.ti,ab,kw
10. 1 OR 2 OR 3 OR 4 OR 5 OR 6 OR 7 OR 8 OR 9
11. metabolic syndrome.ti,ab,kw
12. MS.ti,ab,kw
13. MetS.ti,ab,kw
14. syndrome X.ti,ab,kw
15. cardiometabolic risk factor.ti,ab,kw
16. insulin resistance syndrome.ti,ab,kw
17. 11 OR 12 OR 13 OR 14 OR 15 OR 16
18. 10 AND 17

**Search terms for CINAHL through EBSCO (search data:May 1, 2020)**

("sleep duration" OR “sleep hour" OR "sleeping hour" OR "hours of sleep" OR "sleep time" OR "sleep length" OR "sleep period" OR "sleeping time" OR "sleep span") AND (“metabolic syndrome” OR “MetS” OR “MS” OR “syndrome X” OR “cardiometabolic risk factor” OR “insulin resistance syndrome”)

**Search terms for PsycINFO through Ovid (search data:May 1, 2020)**

1. sleep duration.tw
2. sleep hour.tw
3. sleeping hour.tw
4. hours of sleep.tw
5. sleep time.tw
6. sleep length.tw
7. sleep period.tw
8. sleeping time.tw
9. sleep span.tw
10. 1 OR 2 OR 3 OR 4 OR 5 OR 6 OR 7 OR 8 OR 9
11. metabolic syndrome.tw
12. MS.tw
13. MetS.tw
14. syndrome X.tw
15. cardiometabolic risk factor.tw
16. insulin resistance syndrome.tw
17. 11 OR 12 OR 13 OR 14 OR 15 OR 16
18. 10 AND 17
