# Supplementary material for: Sleep Duration and the Risk of Metabolic Syndrome in Adults: A Systematic Review and Meta-Analysis
Source: Front Neurol. 2021 Feb 18;12:635564. doi: 10.3389/fneur.2021.635564 (PMC7935510; doi:10.3389/fneur.2021.635564)
Supplement: Supplementary file 4 [file Data_Sheet_2.docx]

install.packages("metafor")

install.packages("readxl")

# import data_____________________________________________

library(readxl)

library(metafor)

getwd()

shortall<-read_excel("C:/Users/HuaJianian/Desktop/MS meta/all.xlsx",sheet="森林图short")

shortall$yi=log(shortall$OR)

shortall$xi=(log(shortall$CIUL)-log(shortall$CILL))/3.92

shortcross<-subset(shortall,studytype=="cross-sectional")

shortcohort<-subset(shortall,studytype=="cohort")

longall<-read_excel("C:/Users/HuaJianian/Desktop/MS meta/all.xlsx",sheet="森林图long")

longall$yi=log(longall$OR)

longall$xi=(log(longall$CIUL)-log(longall$CILL))/3.92

longcross<-subset(longall,studytype=="cross-sectional")

longcohort<-subset(longall,studytype=="cohort")

#亚组分析单位————————————————————————————————————————————————————

meta1<-rma(yi, data=shortcross,sei=xi,measure="GEN",method="DL",subset=单位=="community")

meta2<-rma(yi, data=shortcross,sei=xi,measure="GEN",method="DL",subset=单位=="hospital")

meta3<-rma(yi, data=shortcross,sei=xi,measure="GEN",method="DL",subset=单位=="company or office")

summary(meta1)

summary(meta2)

summary(meta3)

predict(meta1,transf=exp, digits=2)

predict(meta2,transf=exp, digits=2)

predict(meta3,transf=exp, digits=2)

#节约工序，手动改一改——————————————————————————————————

diffes<-coef(meta1)-coef(meta3)

diffesse<-sqrt((meta1$se)^2+(meta3$se)^2)

diffesll<-diffes-1.96*diffesse

diffesul<-diffes+1.96*diffesse

zvalue<-diffes/diffesse

pvalue<-round(2*pnorm(-abs(zvalue)),4)

pvalue

#亚组测量方法————————————————————————————————————————————————————

meta2<-rma(yi, data=shortcross,sei=xi,measure="GEN",method="DL",subset=睡眠定义=="interview")

meta1<-rma(yi, data=shortcross,sei=xi,measure="GEN",method="DL",subset=睡眠定义=="questionnaire")

meta3<-rma(yi, data=shortcross,sei=xi,measure="GEN",method="DL",subset=睡眠定义=="sq")

meta4<-rma(yi, data=shortcross,sei=xi,measure="GEN",method="DL",subset=睡眠定义=="objective")

summary(meta1)

summary(meta2)

summary(meta3)

summary(meta4)

predict(meta1,transf=exp, digits=2)

predict(meta2,transf=exp, digits=2)

predict(meta3,transf=exp, digits=2)

predict(meta4,transf=exp, digits=2)

#节约工序，手动改一改——————————————————————————————————

diffes<-coef(meta1)-coef(meta4)

diffesse<-sqrt((meta1$se)^2+(meta4$se)^2)

diffesll<-diffes-1.96*diffesse

diffesul<-diffes+1.96*diffesse

zvalue<-diffes/diffesse

pvalue<-round(2*pnorm(-abs(zvalue)),4)

pvalue

#亚组定义睡眠————————————————————————————————————————————————————

meta2<-rma(yi, data=shortcross,sei=xi,measure="GEN",method="DL",subset=MS=="NECP")

meta1<-rma(yi, data=shortcross,sei=xi,measure="GEN",method="DL",subset=MS=="modified")

meta3<-rma(yi, data=shortcross,sei=xi,measure="GEN",method="DL",subset=MS=="AHA")

meta4<-rma(yi, data=shortcross,sei=xi,measure="GEN",method="DL",subset=MS=="IDF")

meta5<-rma(yi, data=shortcross,sei=xi,measure="GEN",method="DL",subset=MS=="other")

summary(meta1)

summary(meta2)

summary(meta3)

summary(meta4)

summary(meta5)

predict(meta1,transf=exp, digits=2)

predict(meta2,transf=exp, digits=2)

predict(meta3,transf=exp, digits=2)

predict(meta4,transf=exp, digits=2)

predict(meta5,transf=exp, digits=2)

#节约工序，手动改一改——————————————————————————————————

diffes<-coef(meta2)-coef(meta5)

diffesse<-sqrt((meta2$se)^2+(meta5$se)^2)

diffesll<-diffes-1.96*diffesse

diffesul<-diffes+1.96*diffesse

zvalue<-diffes/diffesse

pvalue<-round(2*pnorm(-abs(zvalue)),4)

pvalue

#亚组分析评分————————————————————————————————————————————————————

meta1<-rma(yi, data=longcross,sei=xi,measure="GEN",method="DL",subset=quality=="3")

meta2<-rma(yi, data=longcross,sei=xi,measure="GEN",method="DL",subset=quality=="1")

summary(meta1)

summary(meta2)

predict(meta1,transf=exp, digits=2)

predict(meta2,transf=exp, digits=2)

#节约工序，手动改一改——————————————————————————————————

diffes<-coef(meta2)-coef(meta1)

diffesse<-sqrt((meta2$se)^2+(meta1$se)^2)

diffesll<-diffes-1.96*diffesse

diffesul<-diffes+1.96*diffesse

zvalue<-diffes/diffesse

pvalue<-round(2*pnorm(-abs(zvalue)),4)

pvalue

#亚组分析时间————————————————————————————————————————————————————

meta1<-rma(yi, data=shortcross,sei=xi,measure="GEN",method="DL",subset=short=="5")

meta2<-rma(yi, data=shortcross,sei=xi,measure="GEN",method="DL",subset=short=="6")

meta3<-rma(yi, data=shortcross,sei=xi,measure="GEN",method="DL",subset=short=="7")

meta4<-rma(yi, data=shortcross,sei=xi,measure="GEN",method="DL",subset=short=="8")

summary(meta1)

summary(meta2)

summary(meta3)

summary(meta4)

predict(meta1,transf=exp, digits=2)

predict(meta2,transf=exp, digits=2)

predict(meta3,transf=exp, digits=2)

predict(meta4,transf=exp, digits=2)

#节约工序，手动改一改——————————————————————————————————

diffes<-coef(meta1)-coef(meta4)

diffesse<-sqrt((meta1$se)^2+(meta4$se)^2)

diffesll<-diffes-1.96*diffesse

diffesul<-diffes+1.96*diffesse

zvalue<-diffes/diffesse

pvalue<-round(2*pnorm(-abs(zvalue)),4)

pvalue

#start meta_____________________________________________

metashortcross<-rma(yi, data=shortcross,sei=xi,measure="GEN",method="DL")

metashortcohort<-rma(yi, data=shortcohort,sei=xi,measure="GEN",method="DL")

metalongcohort<-rma(longcohort$yi, data=longcohort,sei=longcohort$xi,measure="GEN",method="DL")

metalongcross<-rma(longcross$yi, data=longcross,sei=longcross$xi,measure="GEN",method="DL")

predict(metalongcohort, digits=2)

predict(meta1,transf=exp, digits=2)

predict(meta2,transf=exp, digits=2)

predict(meta3,transf=exp, digits=2)

summary(metalongcohort)

summary(meta2,transf=exp)

summary(meta3,transf=exp)

#trim and fill

trimfill(metashortcohort,transf=exp)

rtf<-trimfill(metashortcohort)

funnel(rtf)

trimfill(metashortcross,transf=exp)

rtf<-trimfill(metashortcross)

funnel(rtf)

trimfill(metalongcohort)

rtf<-trimfill(metalongcohort)

funnel(rtf)

trimfill(metalongcross)

rtf<-trimfill(metalongcross)

funnel(rtf)
